# Plasma proteomic study of acute mountain sickness susceptible and resistant individuals

**DOI:** 10.1038/s41598-018-19818-9

**Published:** 2018-01-19

**Authors:** Hui Lu, Rong Wang, Wenbin Li, Hua Xie, Chang Wang, Ying Hao, Yuhuan Sun, Zhengping Jia

**Affiliations:** Key Laboratory of the plateau of environmental damage control, Lanzhou General Hospital of Lanzhou Military Command, No. 333 Binhe South Road, Lanzhou 730050 Gansu, China

## Abstract

Although extensive studies have focused on the development of acute mountain sickness (AMS), the exact mechanisms of AMS are still obscure. In this study, we used isobaric tags for relative and absolute quantitation (iTRAQ) proteomic analysis to identify novel AMS−associated biomarkers in human plasma. After 9 hours of hypobaric hypoxia the abundance of proteins related to tricarboxylic acid (TCA) cycle, glycolysis, ribosome, and proteasome were significantly reduced in AMS resistant (AMS−) group, but not in AMS susceptible (AMS+) group. This suggested that AMS− individuals could reduce oxygen consumption via repressing TCA cycle and glycolysis, and reduce energy consumption through decreasing protein degradation and synthesis compared to AMS+ individuals after acute hypoxic exposure. The inflammatory response might be decreased resulting from the repressed TCA cycle. We propose that the ability for oxygen consumption reduction may play an important role in the development of AMS. Our present plasma proteomic study in plateau of the Han Chinese volunteers gives new data to address the development of AMS and potential AMS correlative biomarkers.

## Introduction

Acute mountain sickness (AMS) is a transient medical condition characterized by headache, nausea, fatigue, dizziness and insomnia that usually occur within 6–12 h after rapid ascent to high altitudes above 2500 m in non-altitude acclimatized individuals^1^. AMS is less severe but has much higher incidence than the other two major acute high-altitude sicknesses, high-altitude cerebral edema (HACE) and high-altitude pulmonary edema (HAPE)^1,2^.

Although the development of AMS has been extensively studied, the basic pathogenic mechanism of AMS remains an open question. It was generally believed that central nervous system complications were linked to hypoxia and hypoxemia^3^. And it was hypothesized that hypoxia induced series of neurohumoral and hemodynamic responses, including inflammatory response, disruption of blood-brain barrier (BBB), nitric oxide (NO)-mediated activation of trigeminovascular system, and inadequate venous drainage capacity for elevated venous drainage requirements may be involved in the pathophysiology of AMS^3–7^. Genetic and proteomic studies showed that certain biomarkers might be involved in AMS development. The levels of these biomarkers are different in AMS susceptible (AMS+) individuals and AMS resistant (AMS−) individuals^5,8^. Therefore, it is helpful to explore the AMS−associated biomarkers for addressing the mechanism of AMS. However, the understanding of AMS−associated biomarkers is still insufficient in-depth. Proteomics method can be used to analyze plasma samples from AMS− and AMS+ individuals to find the AMS−associated biomarkers. A broad view provided by global proteomics can provide novel insights into the etiology of AMS and identify new avenues for the prediction, prevention, and treatment of AMS. In the present study, we compared the changes in proteome of plasma samples between AMS+ group and AMS− group after an acute exposure to high altitudes. Our study suggested that AMS− individuals might be more capable to down-regulate oxygen consumption than AMS+ individuals after acute hypobaric hypoxic exposure.

## Results

### Characteristics of subjects

**Figure 1:**
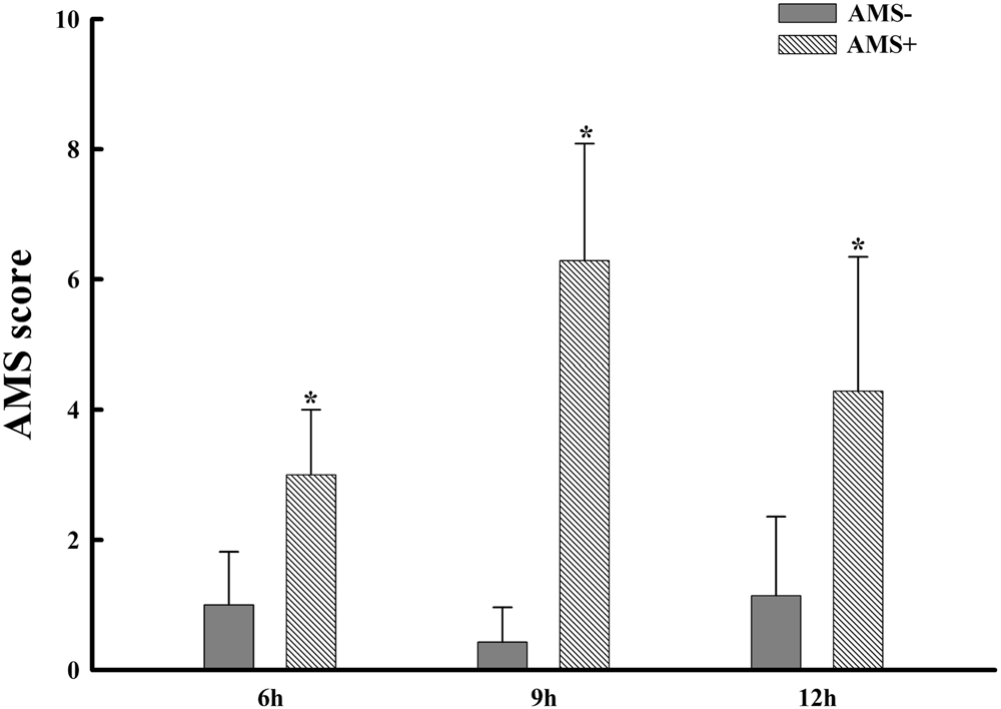
LLQ scores were lower in the AMS− group than the AMS+ group at 6 hours, 9 hours and 12 hours of exposure to the altitude of 3800 m. Stars represent p < 0.05 AMS− group versus AMS+ group.

Age (28.0 ± 3.3 years *vs*. 27.4 ± 1.5 years, p = 0.682), weight (69.0 ± 12.5 kg *vs*. 61.4 ± 13.2 kg, p = 0.291), height (170.3 ± 7.8 cm *vs*. 167.4 ± 9.0 cm, p = 0.536) and body mass index (BMI) (23.6 ± 2.7 kg/m^2^ *vs*. 21.7 ± 2.3 kg/m^2^, p = 0.168) were similar between AMS− and AMS+ groups. LLQ scores were lower in the AMS− group than the AMS+ group at 6 hours (1.00 ± 0.82 *vs*. 3.00 ± 1.00, p < 0.05), 9 hours (0.43 ± 0.53 *vs*. 6.29 ± 1.80, p < 0.05) and 12 hours (1.14 ± 1.22 *vs*. 4.29 ± 2.06, p < 0.05) of exposure to the altitude of 3800 m (Fig. 1).

### AMS+ group global proteomic responses to acute high-altitude exposure

To investigate the global proteins changes in plasma in AMS+ subjects after acute exposure to high altitude, iTRAQ proteomic analysis was used to identify protein profiles of the BL status (AMS + BL) (low altitude of 400 m) and 9 h after exposure to high altitude (AMS + 9) (high altitude of 3800 m) in AMS+ subjects plasma. A total of 89 proteins were founded differentially expressed due to high-altitude stressor (fold change > 1.5), among which 17 proteins were up-regulated and 72 proteins were down-regulated (see supplementary Table S1). To determine the most prominent functional protein groups that differed in response to hypobaric hypoxia in AMS− subjects, we performed GO analysis and identified that the affected proteins enriched in the metabolic process, cellular process, organic substance metabolic process, single-organism process and so on. In the sections of discussion, we will discuss the potential relevance of these findings for human response to hypobaric hypoxia and AMS.

### AMS− group global proteomics responses to acute high-altitude exposure

To probe the effects of short-term high altitude (3800 m) exposure on protein level changes in the plasma of AMS− individuals, we used iTRAQ approach to identify protein profiles of the BL condition (AMS − BL) and 9 h after in a short-time hypobaric hypoxia (AMS − HA) in AMS− plasma. A total of 421 differentially expressed proteins were observed (fold change > 1.5), among which 61 proteins were up-regulated and 360 proteins were down-regulated (see supplementary Table S1). To reveal the most prominent functional protein groups that differed in response to hypobaric hypoxia in AMS− individuals, GO biological process analysis was performed, and we found that the affected proteins were involved in cellular process, metabolic process, organic substance metabolic process, and primary metabolic process and so on. It was noted that 10 pivotal metabolic enzymes of TCA cycle, pyruvate dehydrogenase alpha 1 (PDHA1), dihydrolipoyl dehydrogenase (DLD), ATP-citrate synthase (ACLY), aconitase (ACON), isocitrate dehydrogenase (IDH1, IDH2, IDH3A, IDH3B), 2-oxoglutarate dehydrogenase (OGDH), dihydrolipoyllysine-residue succinyltransferase (DLST), succinyl-CoA ligase (SUCLG1,SUCLG2, SUCLA2), succinate dehydrogenase (SDHA, SDHB), and malate dehydrogenase (MDH2), were markedly decreased in AMS− group after acute exposure to high altitude. Besides, a significant decrease of the proteins related to glycolysis, ribosome and proteasome pathway had also been observed in AMS− group after acute high-altitude exposure.

### KEGG pathway analysis

We used the KEGG pathway to analyze expression alteration of proteins stressed by exposure to high altitude in AMS+ and AMS− group. After acute exposure to hypobaric hypoxia the different proteins were enriched in 105 pathways, predominant in complement and coagulation cascades, malaria, pertussis, prion diseases, staphylococcus aureus infection and so on for the AMS+ group, and were enriched in 198 pathways, predominant in tricarboxylic acid (TCA) cycle, ribosome, metabolic pathways, glycerolipid metabolism, ascorbate and aldarate metabolism, propanoate metabolism, proteasome etc. for the AMS− group (see supplementary Table S2).

## Discussion

Identifying the proteins involved in physiological and pathological processes associated with hypoxia would shed light on the mechanisms of diseases caused by lack of oxygen, such as AMS. Our present plasma proteomic study in plateau of the Han Chinese volunteers gives new data to address the development of AMS and potential AMS correlative biomarkers. In the present study, we test the plasma proteome of AMS− and AMS+ individuals in BL and short-term (9 hours) high-altitude exposure status. We found that proteins response to acute hypobaric hypoxic exposure in AMS+ individuals and AMS− individuals were different using iTRAQ proteomic analysis. These different proteins may help to address the pathology of AMS.

Despite the fact that many theories explaining the development of AMS have been proposed during the past decades, the basic pathogenic mechanism of AMS is still fairly unclear. The blood brain barrier (BBB) theory, one of those hypotheses, suggests that hypoxia-induced hypoxemia will initiate an inflammatory response with the release of inflammatory mediators that contribute to an increase of the capillary pressure by over perfusion and vasodilatation, and elevate the permeability of the BBB by disrupting the BBB. This increases the potential for capillary leak and cerebral edema, which in turn causes the traction of the meninges and blood vessels, and high-altitude headache^9–11^. Recently, some studies suggested that reactive oxygen species (ROS) formed during short-term hypoxia exposure and might be involved in cerebral vascular leak and astrocyte swelling in the trigeminal areas which play a crucial role in causing AMS^12^. According to present evidences, inflammation response and antioxidant response may play important roles in developing AMS. The mechanism of AMS is not very clear yet, but increased consumption of oxygen must aggravate the symptoms of AMS at the high altitude.

**Figure 2:**
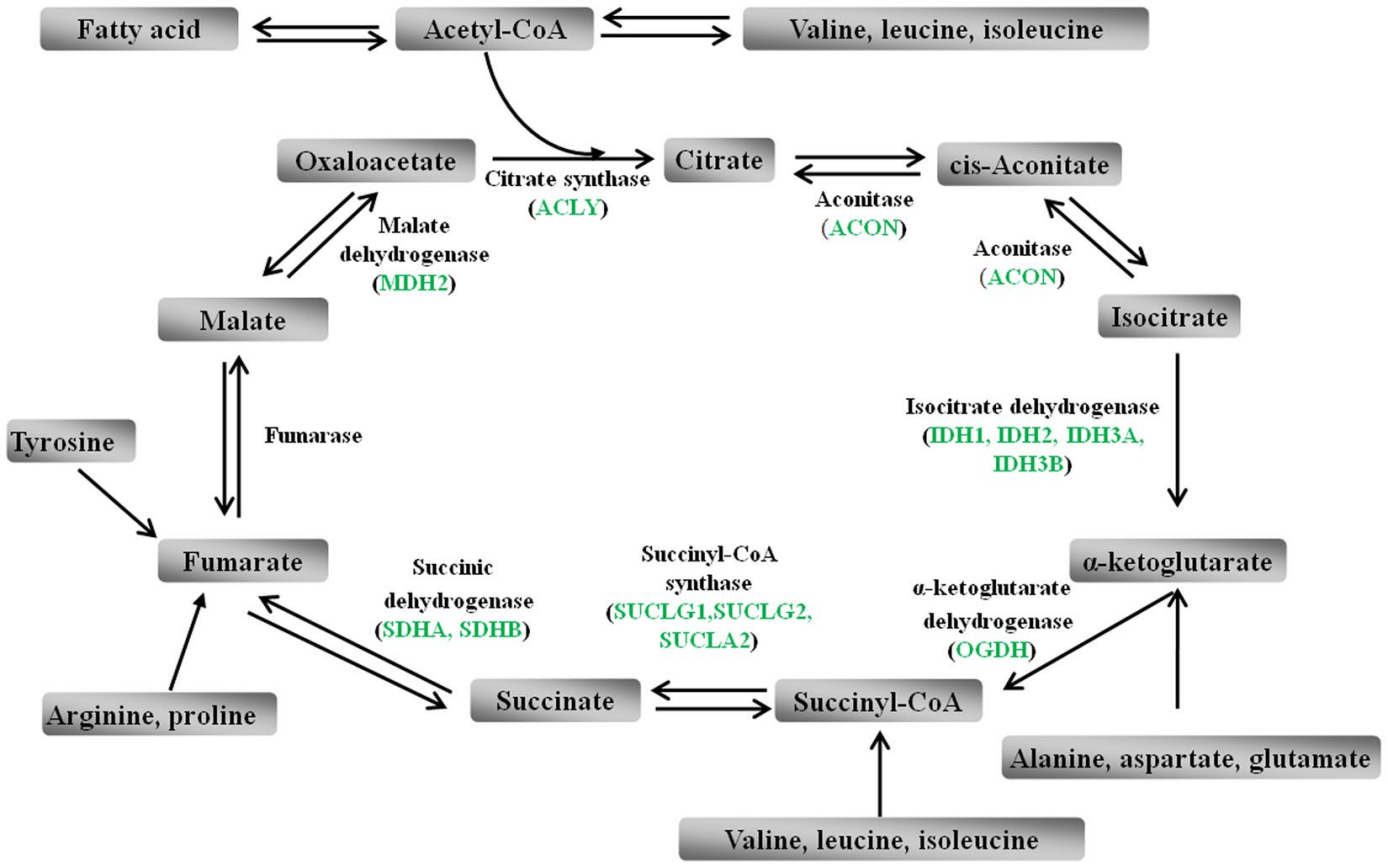
Levels of 10 pivotal metabolic enzymes of TCA cycle, including PDHA1, DLD, ACLY, ACON, IDH1/IDH2/IDH3A/IDH3B, OGDH, DLST, SUCLG1/SUCLG2/SUCLA2, SDHA/SDHB, and MDH2, were markedly decreased in AMS− group after acute exposure to high altitude. Green color represents down-regulated levels.

After exposure to hypoxia, metabolic adaptation is essential for normal tissue functions. In cell models, maintaining oxygen concentration lays in a fast and reversible metabolic switch from oxidative metabolic process to anaerobic glycolytic process^13–15^. In human body, the molecular mechanism of the adaptive response to hypoxia is still not well known. TCA cycle is a crucial oxidative metabolic process in mitochondria to produce energy. Our proteomic analyses showed that levels of 10 pivotal metabolic enzymes of TCA cycle, including PDHA1, DLD, ACLY, ACON, IDH1/IDH2/IDH3A/IDH3B, OGDH, DLST, SUCLG1/SUCLG2/SUCLA2, SDHA/SDHB, and MDH2, were markedly decreased in AMS− group after acute exposure to high altitude (Fig. 2). While only SUCLG2 was down-regulated in AMS+ group after acute exposure to hypobaric hypoxia. These results are consistent with the recent proteomic study with muscle, which showed that both TCA cycle and glycolysis were repressed after exposure to hypobaric hypoxia^22^.

Low-level-oxygen is undoubtedly a trigger of AMS, it is reported that increased consumption of oxygen at the high altitude aggravated the symptoms of AMS^16^. Thus, the repressed TCA cycle in AMS− individuals after acute exposure to a high altitude of 3800 m, suggested a decreased consumption of oxygen, may be one of the factors of resistance to AMS. Moreover, as a pivotal metabolic process, TCA cycle provides precursors for certain amino acids and fatty acid synthesis, and intermediates of TCA cycle can be metabolized into amino acids and fatty acid and vice versa (Fig. 2). In the present study the alteration of several amino acids (valine, leucine, isoleucine, lysine, arginine, proline, alanine, aspartate and glutamate) and fatty acid could be explained (at least partly) by the suppressed TCA cycle. These results indicated that AMS− individuals could regulate the efficacy of TCA cycle to reduce the consumption of oxygen when exposure to hypobaric hypoxia, but AMS+ individuals could not. It seems that the minor ability of AMS+ individuals to reduce its oxygen consumption make them more dependent of stable and relatively high level of environmental oxygen, which contribute to their higher AMS susceptibility. But further research will be needed to confirm this point.

**Figure 3:**
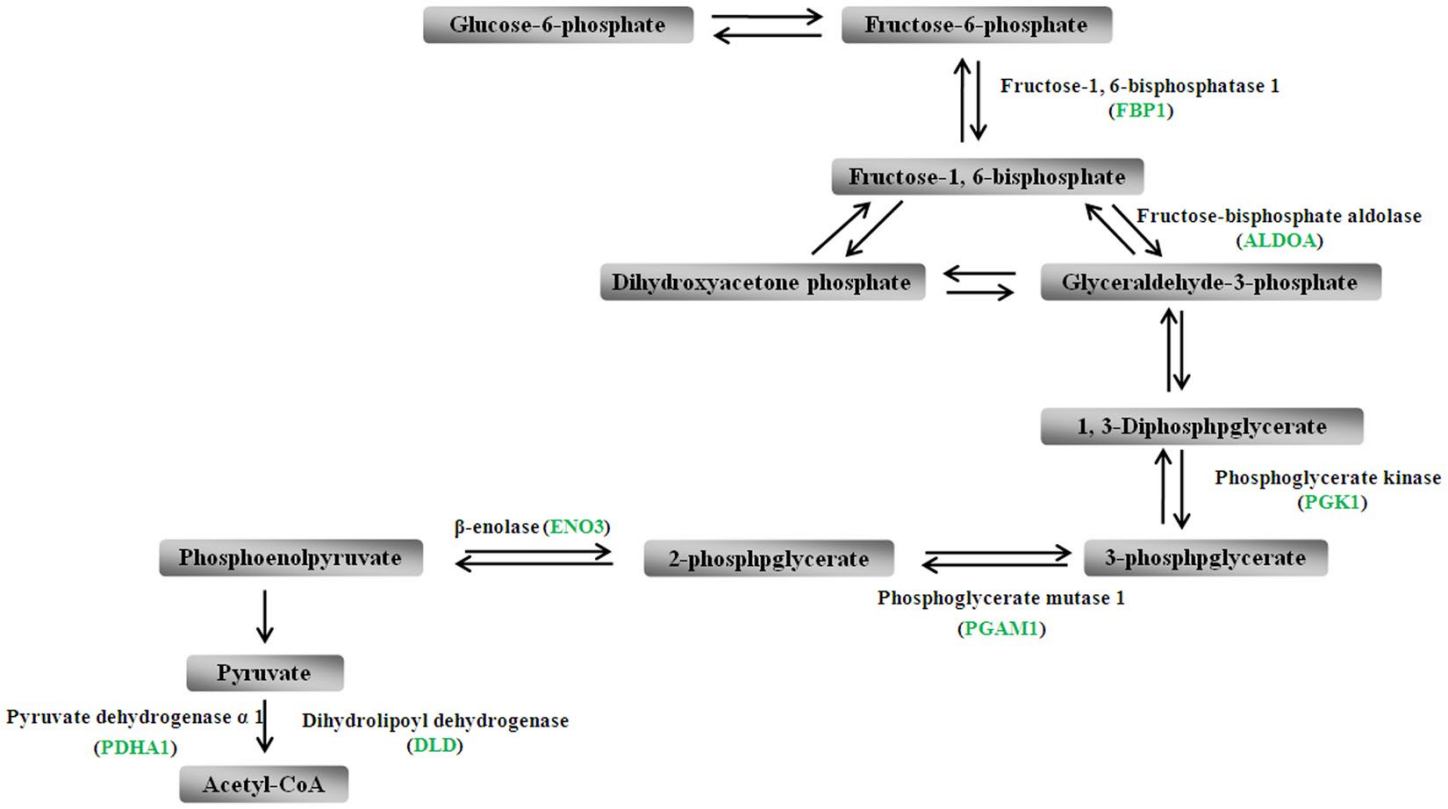
Levels of 10 crucial metabolic intermediates of GALM, FBP1, ALDOA, PGK1, PGAM1, ENO3, PDHA1, DLD, AKR1A1, and ALDH1B/ALDH7A1/ALDH8A1/ALDH9A1, were significantly decreased in AMS− group after short time hypoxia exposure. Green color represents down-regulated levels.

In the other hand, some intermediates of the TCA cycle were identified as inflammatory signals. For instance, succinate, a metabolic intermediate of the TCA cycle, playing an important role in adenosine triphosphate (ATP) generation^17^, has been recently proposed for a novel function apart from the TCA cycle. Succinate activates hypoxia-inducible factor-1α (HIF-1α) through inhibiting prolyl hydroxylase domain (PHD) enzyme function and enhancing ROS production^18^. Subsequently, succinate enhances glycolytic process via elevated HIF-1α which is a transcriptional regulator in the switch to glycolysis and increased ROS which inhibits mitochondrial function, in turn boosting glycolysis as a result^17,19^. In our present study, levels of 10 crucial metabolic intermediates of glycolysis, aldose 1-epimerase (GALM), fructose-1,6-bisphosphatase 1 (FBP1), fructose-bisphosphate aldolase (ALDOA), phosphoglycerate kinase (PGK1), phosphoglycerate mutase 1 (PGAM1), beta-enolase (ENO3), pyruvate dehydrogenase alpha 1 (PDHA1), dihydrolipoyl dehydrogenase (DLD), alcohol dehydrogenase (AKR1A1), and aldehyde dehydrogenase (ALDH1B, ALDH6A1, ALDH7A1, ALDH8A1), were significantly decreased in AMS− group after short-time of hypoxia exposure (Fig. 3). The repressed glycolysis may be caused by inhibiting TCA cycle, especially by the decreases of succinate (attenuate SUCLG1, SUCLG2, SUCLA2, SDHA, and SDHB). And then, the reduced ROS production and HIF-1α accumulation may be causes of resistance to AMS in AMS− individuals after rapid ascent to high altitude. However, further in-depth studies are needed to confirm this point.

Cytosolic citrate is required for fatty acid biosynthesis and metabolized to produce reactive oxygen intermediates and prostaglandins, important inflammatory mediators^20^. Our present results showed that ATP-citrate synthase (ACLY) was decreased, suggesting that the decreases of citrate may in turn inhibit the inflammatory process. Furthermore, the fatty acid biosynthesis (fatty acid synthase, FASN) may be decreased in plasma samples of AMS− individuals, which may be partly resulted from the citrate reduction. The decreased level of citrate would lead to NO and ROS reduction, in turn decreasing hypoxia induced oxidative damage.

Nicotinamide adenine dinucleotide (NAD^+^) exerts several anti-inflammatory effects by activating sirtuins (Sirts), a class of NAD^+^-dependent deacetylases. Sirt1 inhibits the proinflammatory transcription factors nuclear factor kB (NF-kB), activator protein 1 (AP-1), HIF-1α, and glycolysis^21–24^. Sirt6 impairs NF-КB-dependent signal pathway by deacetylating target promoters^25^. The levels of NAD^+^ would be increased by attenuating isocitrate dehydrogenase (IDH1, IDH2, IDH3A, IDH3B), 2-oxoglutarate dehydrogenase (OGDH), and malate dehydrogenase (MDH2) in AMS− individuals after acute exposure to hypobaric hypoxia (Fig. 2). As discussed above, the increased NAD^+^ could inhibit proinflammatory NF-КB signal pathway, which was suppressed (IGHA2, IGHA1, CPNE3, IGHG1, IGHM, tr|A2N011|A2N011_HUMAN) in AMS−individuals in our present study. Coupled with these results, we assumed that the metabolic intermediates of TCA cycle involved in inflammatory process may play an important role in AMS development.

Protein synthesis is a fundamental cell process of ribosome. Ribosome is the site for biosynthesis which cost lots of energy and oxygen, assigned to the task of decoding the genetic code. There are considerable evidence showing that hypoxia can significantly affect the ribosome assembly and proteins synthesis^26,27^. After acute exposure to high altitude of 3800 m, the ribosome biosynthesis pathway was markedly down-regulated in AMS− group, but not in AMS+ group. Reduced ribosome means attenuated ribosome assembly and protein synthesis, lead to less ATP and oxygen consumption. Consisted with this, in the AMS− group, the proteasome pathway (involving PSMC3, PSMA3, PSMD12, PSMD1, PSMD2, PSMD11, PSMA7, PSMC4, PSMB1, PSMA2, PSMA1, PSMC5, PSMA6, PSMD7) was also greatly suppressed, suggesting that the life of proteins is prolonged and the proteins biosynthesis is less needed. In contrary, after acute exposure to hypoxia, only four ribosomal proteins (Rps2p, Rps9p, Rps24p, Rpl15p) are significantly decreased in AMS+ group. These results demonstrated that reduced ribosome assembly and prolonged protein life potentially decreased oxygen consumption, which might be involved in the resistance to AMS of AMS− individuals.

The exact mechanism of ROS functioning in AMS is not very clear yet. Many studies showed that the hypoxia could increase the production of ROS *in vivo* and suggested that ROS and other oxidative proteins might play important roles in causing AMS. Line of evidence suggested that ROS was formed during acute exposure to hypoxia and contributed to cerebral vascular leak and astrocyte swelling in the trigeminal areas^12^. This has led some researchers to hypothesize that hypoxia increase the production of ROS in the brain, which were subsequently responsible for developing AMS^28–30^. Unfortunately, however, exogenous antioxidant (L-ascorbic acid, α-tocopherol acetate, and α-lipoic acid) therapy as a strategy to prevent AMS in human has been proved ineffective^31^. One recent plasma proteomic study of AMS in hypoxic chamber showed that antioxidant properties (i.e. peroxiredoxin-6 (Prdx-6), glutathione peroxidase (GPx), and sulfhydryl oxidase 1(QSQX1)) rose in AMS+ individuals but not in AMS− individuals^5^. Their exploratory analyses suggested that enzymatic antioxidant systems were enhanced after exposure to hypobaric hypoxia in AMS+ individuals but not AMS− individuals. They speculated that enhanced antioxidant systems might be overcompensation for hypoxia-induced oxidant production in AMS+ individuals and questioned that oxidant production played an important role in the development of AMS. While, our proteomic analysis results showed that the abundance of proteins related to TCA cycle, glycolysis, ribosome, and proteasome were significantly reduced in AMS− group, but not in AMS+ group after acute ascent to high altitude. We speculate that oxygen consumption, not oxidant production, may play an important role in the development of AMS. Oxidant production, such as ROS, may decrease resulting from less oxygen consumption.

It was noticed that the vascular endothelial growth factor (VEGF) signal pathway was suppressed in the AMS− group after acute hypoxic exposure, but not in the AMS+ group. Many studies have showed that VEGF seems likely to be a biomarker of AMS. VEGF is a critical angiogenic factor and hypoxia-induced protein^32,33^ that can increase vascular permeability^34,35^. Blocking VEGF can prevent hypoxic brain edema^36^. Although in the present study we did not observed changes in VEGF, we found that CEPN3, which is a key regulator of VEGF signal pathway, is significantly decreased after exposure to hypoxia in AMS− individuals, but not in AMS+ individuals. Coupled with previous studies, we assume that VEGF signal pathway may participate in the development of AMS.

**Figure 4:**
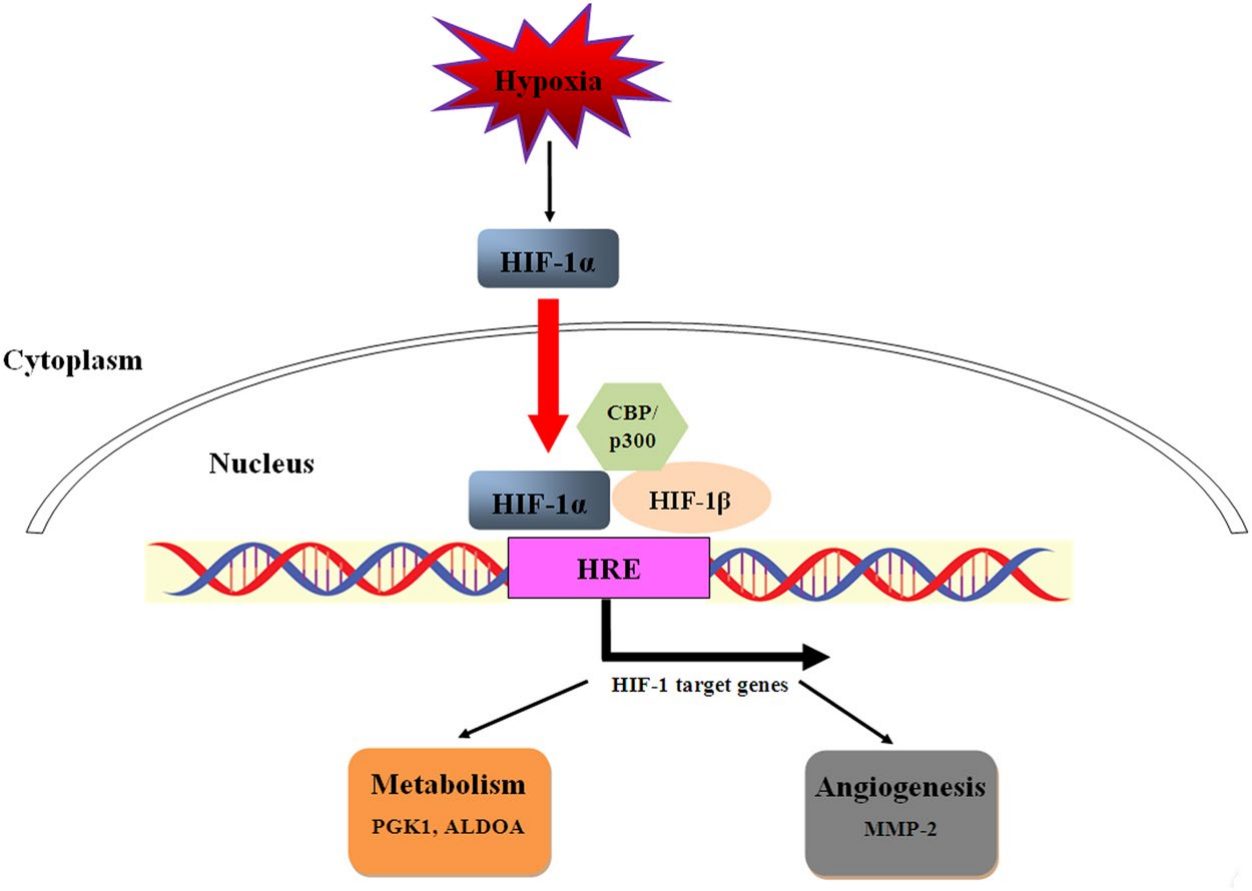
Hypoxia regulated HIF signal pathway. Our present proteomic results showed that ALDOA, PGK1, and MMP-2 were down-regulated in AMS− individuals after acute exposure to hypoxia, but not AMS+ individuals.

One recent study showed that Tibetans have a hyporesponsive HIF signal pathway and blunted physiological responses to hypoxia living at sea level^37^. It is hinted that HIF signal pathway may be involved in the development of AMS. Several studies demonstrated that ALDOA^38^, PGK1^39^ and MMP-2^40^ are targets of HIF-1 signal pathway. Our present proteomic results showed that ALDOA, PGK1, and MMP-2 were down-regulated in AMS− individuals after acute exposure to hypoxia, but not in AMS+ individuals (Fig. 4). Our present study demonstrated that HIF-1 signal pathway may be repressed in AMS− individuals after rapid ascent to high altitude, but further researches are needed to confirm this point.

### Study Limitations

Plasma from peripheral blood samples may not accurately reflect the cerebral environment. However, peripherally circulating proteins can affect cerebral endothelial permeability^41^, so making possible to evaluate AMS susceptibility through plasma proteomic analysis. Considering ethical limitations and participants safety, peripheral blood sample collection was unique choice for AMS biomarker investigation. Secondly, a technical challenge of global plasma proteomics is that the plasma proteome encompasses a larger number of proteins and a more dynamic protein concentration range than any other single human sample^42^. Even though after several depletion steps, it is not possible to detect proteins of very low abundance that may be of importance for AMS. Thirdly, we analyzed pooled samples by mass spectrometry rather than individual samples. Sample pooling reduces biologic variance between specimens in proteomic and microarray analyses^43–46^ and, consequently, facilitates the identification of the most robust differences between groups^43,47^. A comprehensive evaluation of the effect of sample pooling for proteomic analysis demonstrated that, for the majority of proteins, data obtained from pooled samples accurately represented the mean protein levels of individual samples that composed the pool rather than being skewed by one or two samples^43^. Although reducing biological variation masks unique features of individual samples that compose the sample pool, this strategy is advantageous when the research focus is to elucidate common features of a given population or disease group^45^, as was the case in our study. Further supporting the validity of the proteomic methods we used, a recent paper confirmed that differentially expressed proteins identified by mass spectrometry in high-altitude natives versus sea-level controls were consistent with the results obtained by Western blot and enzyme-linked immunosorbent assay^48^. Finally, although our work has provided some important clues, we do not make a further verification of our proteomic results on whole animal or cell culture models. The primary reason is subjective symptoms of AMS. Coupled with the elusive mechanisms of AMS, especially high altitude headache, we cannot model the AMS in animals. To further test the proteomic results in human we need numerous samples but this implies insurmountable ethical difficulties.

## Conclusions

Our present study found that proteins related to TCA cycle, glycolysis, ribosome, and proteasome decreased in AMS− individuals after acute exposure to high altitude, but not in AMS+ individuals. These results suggested that AMS− individuals could reduce oxygen consumption via repressing TCA cycle and glycolysis, and reduce energy consumption through decreasing protein degradation and synthesis compared to AMS+ individuals. The inflammatory response might be decreased resulting from the repressed TCA cycle. We speculate that oxygen consumption may play an important role in the development of AMS. However, further researches are needed to confirm this point.

## Materials and Methods

### Ethics statement

This study has been approved by the Institutional Review Board (IRB) of Lanzhou General Hospital of Lanzhou Military Command (Lanzhou, China). The use of human plasma samples for research purpose was authorized by the IRB of Lanzhou General Hospital of Lanzhou Military Command. All the volunteers agreed to participate in this study with signed informed consent document (Supplementary Information). All experiments were performed in accordance with relevant guidelines and regulations.

### Subjects

We recruited 23 local Chinese volunteers aged 25 to 35 years. They primarily resided at an elevation of 400 m or lower in the area of Xi’an, China. Recruitment, screening and exclusion criteria used in this study were as described previously^5,49^. Briefly, screening procedures to assess general health status and to determine eligibility prior to participation included medical history check, physical and neurological exam, standard blood and urine analysis, and a maximal exercise test. We excluded those who 1) had any health problems; 2) had an abnormal complete blood count, chemistry panel, or liver function results; 3) were pregnant or intended to become pregnant within the near future; 4) history of migraine, headache, seizure, or head injury with loss of consciousness; 5) with mechanical limitations or metal implants that prohibited exercise; 6) were smoker; 7) were regular on prescription medications; inability to reach a workload of least 200 W during the incremental exercise test; 8) or altitude exposure greater than 2500 m within three months of study. All subjects in each group were the Han Chinese and not related.

### Study design and ascent profile

Peripheral venous blood samples were collected and AMS symptoms were evaluated at low altitude (400 m; Xi’an, China) 24 hours (baseline, BL) in advance. To acutely expose volunteers to high altitude, all subjects were transported to the altitude of 3800 m (Yushu, China) from Xi’an (400 m) in 3 hours by flight. AMS symptoms of subjects were evaluated immediately when landed. This is time point 0 h. The following studies were conducted in Yushu Bayi Hospital, which has a phase I clinical trial qualification and can treat high-altitude illness. Two hours after exposure to the altitude of 3800 m, all subjects completed three 5-minute sub-maximal exercise bouts on a cycle with 15 minutes rest in between in the ward. This is to increase the likelihood of developing AMS^16^. The whole process was monitored by one doctor and two nurses.

### Evaluation of AMS status

To evaluate AMS status, subjects completed self-reported sections of the Lake Louise Questionnaire (LLQ) prior to exposure to the 3800 m high altitude (BL and 0 hour) as well as after 3, 6, 9 and 12 hours of hypobaric hypoxia. Subjects were asked to quantify their degree of headache, gastrointestinal upset, fatigue, and dizziness on a four-point scale (no symptoms = 0, mild = 1, moderate = 2, severe = 3). Subjects with a cumulative LLQ score equal or greater than 3 with headache after ascent to high altitude within 6 to 12 hours were defined as AMS+ individuals. Those with the sum of LLQ score equal or less than 2 or without headache after exposure to hypobaric hypoxia were considered as AMS− individuals^50^.

### Plasma collection and sample preparation

Plasma collection and sample preparation was performed as described previously^5^. Blood samples were collected in a semi-recumbent position from an antecubital vein by an indwelling intravenous cannula (BL and 9 hours), and then placed in EDTA-coated blood collection tubes. The plasma was separated from blood cells by centrifugation and stored in 0.2 mL aliquots at −80 °C until analysis.

### Protein preparation

The set of 23 subjects was ranked according to the LLQ scores after exposure to 3800 m in 6 to 12 hours. The top 7 individuals with the highest LLQ scores were defined as the subset of AMS−. The bottom 7 individuals with the lowest LLQ scores were regarded as the AMS+ group. Although the criteria reduced sample size, it maximized the differences between AMS− and AMS+ individuals, and avoided the inclusion of subjects with ambiguous AMS status^4,5^. A series of preprocessing steps, which increased the detection sensitivity of expressions of hypoxia-induced proteins changes in AMS+ group and AMS− group, were implemented before analyzing the plasma samples. First of all, plasma samples collected from the 7 AMS− subjects and 7 AMS+ subjects were pooled at each time point respectively. This resulted in four pooled samples for analysis (two at BL for AMS− and AMS+ groups, and other two at 9 hours for the two groups). Secondly, to reduce the complexity of samples and facilitate the identification of lower-abundance proteins, the highly abundant proteins were depleted using ProteoMinerTM Kits (Bio-Rad Laboratories, Hercules, CA, USA)^51^ after pooling the samples. Samples were eluted in Lysis buffer (7 M Urea, 2 M Thiourea, 4% CHAPS, 40 mM Tris-HCl, pH = 8.5), reduced with 10 mM DTT at 56 °C for 1 h, and followed by alkylation with 55 mM IAM in a darkroom for 1 h. The reduced and alkylated protein mixtures were precipitated by adding 4 fold volume of pre-chilled acetone at -20 °C overnight. After centrifugation at 30,000 g at 4 °C, the pellet was dissolved in 0.5 M TEAB (Applied Biosystems, Milan, Italy) and sonicated in ice. The solution was afterwards centrifuged at 30,000 g at 4 °C, and an aliquot of the supernatant was taken for determination of protein concentration by Bradford protein assay method^52,53^. The remaining supernatant was kept at −80 °C for further analysis.

### Proteomic analysis

One hundred micrograms of total protein were taken out of each sample and the proteins were digested with Trypsin Gold (Promega, Madison, WI, USA) (protein: trypsin = 30:1) at 37 °C for 16 h. The samples were then processed according to the protocol attached to 8-plex iTRAQ reagent (Applied Biosystems).

The peptides were subjected to nanoelectrospray ionization followed by tandem mass spectrometry (MS/MS) in a Q EXACTIVE (Thermo Fisher Scientific, San Jose, CA) coupled online to the HPLC. Intact peptides were detected in the Orbitrap at a resolution of 70 000. Peptides were selected for MS/MS using high-energy collision dissociation (HCD) operating mode with a normalized collision energy setting of 27.0; ion fragments were detected in the Orbitrap at a resolution of 17500. A data-dependent procedure that alternated between one MS scan followed by 15 MS/MS scans was applied for the 15 most abundant precursor ions above a threshold ion count of 20000 in the MS survey scan with following dynamic exclusion duration of 15 s with electrospray voltage of 1.6 kV. Automatic gain control (AGC) was used to optimize the spectra generated by the orbitrap. The AGC target for full MS was 3 × 10^6^ and 1 × 10^5^ for MS2. For MS scans, the *m/z* scan range was 350 to 2000 Da. For MS2 scans, the *m/z* scan range was 100–1800 Da.

### Data analysis

Raw data acquired from the Orbitrap were converted into MGF files using Proteome. Proteins identification was performed by using the Mascot search engine (Matrix Science, London, UK; version 2.3.02) against Uniprot-human database (http://www.uniprot.org) containing 143397 sequences. For protein identification, a mass tolerance of 20 ppm was permitted for intact peptide masses and 0.05 Da for fragmented ions, with allowance for one missed cleavages in the trypsin digests. Gln- > pyro-Glu (N-term Q), Oxidation (M) and deamidated (NQ) are the potential variable modifications. Carbamidomethyl (C), iTRAQ8plex (N-term), and iTRAQ8plex (K) are fixed modifications. To reduce the probability of false peptide identification, only peptides with significance scores (≥20) at the 99% confidence interval by a Mascot probability analysis greater than “identity” were counted as identified. Each confident protein identification involved at least one unique peptide. For protein quantitation, it was required that a protein contained at least two unique peptides. The quantitative protein ratios were weighted and normalized by the median ratio in Mascot. We only used ratios with p-values < 0.05, and only fold changes of >1.5 was considered as significant.

### Functional analysis

Functional annotations of the proteins were conducted using Blast2GO program against the non-redundant protein database (NR; NCBI) . The Kyoto Encyclopedia of Genes and Genomes (KEGG) database (http://www.genome.jp/kegg/)^54^ and the Cluster of Orthologous Groups (COG) database (http://www.ncbi.nlm.nih.gov/COG/) were used to classify and group these identified proteins. Biological processes associated with AMS− and AMS+ individuals were further analyzed by Gene Ontology (GO), which is an international standardization of gene function classification system. KEGG pathway is a collection of manually drawn pathway maps representing our knowledge on the molecular interaction and reaction networks.

### Statistics

Differences in age, height, weight, BMI, and LLQ scores between AMS− and AMS+ individuals were identified by independent sample *t*-tests as appropriate. Data are presented as ‘mean ± standard deviations’. Criteria for significance were set a priori at two-tailed *p* < 0.05.

## Electronic supplementary material

Supplementary table 1

Supplementary table 2
