# Supplementary material for: Plasma proteomic study of acute mountain sickness susceptible and resistant individuals
Source: Sci Rep. 2018 Jan 19;8:1265. doi: 10.1038/s41598-018-19818-9 (PMC5775437; doi:10.1038/s41598-018-19818-9)
Supplement: Supplementary file 1 — Supplementary table 1 [file 41598_2018_19818_MOESM1_ESM.docx]

| Supplementary Table 1. Changes in levels of plasma proteins after acute exposure to high altitude | | | | |
| --- | --- | --- | --- | --- |
| **NO.** | **Proteins** | **Description** | **AMS-HA/ AMS-BL** | **AMS+HA/ AMS+BL** |
| 1 | AADAC | Arylacetamide deacetylase | 0.069 | — |
| 2 | AASS | Alpha-aminoadipic semialdehyde synthase, mitochondrial | 0.083 | 0.684 |
| 3 | ABAT | 4-aminobutyrate aminotransferase, mitochondrial | 0.188 | 0.654 |
| 4 | ACAA2 | acetyl-Coenzyme A acyltransferase 2 (mitochondrial 3-oxoacyl-Coenzyme A thiolase) , nuclear gene encoding mitochondrial protein | — | 0.584 |
| 5 | ACADL | Long-chain specific acyl-CoA dehydrogenase, mitochondrial | 0.078 | 1.077 |
| 6 | ACADM | Medium-chain-specific acyl-CoA dehydrogenase, mitochondrial | 0.010 | — |
| 7 | ACADS | Short-chain acyl-CoA dehydrogenase | — | 0.651 |
| 8 | ACLY | ACLY variant protein (Fragment) | 0.245 | 0.842 |
| 9 | ACON | Aconitase (Fragment) | 0.111 | 1.069 |
| 10 | ACOT13 | Isoform 2 of Acyl-coenzyme A thioesterase 13 | 0.327 | 1.191 |
| 11 | ACP5 | Tartrate-resistant acid phosphatase type 5 (Fragment) | 0.146 | 0.506 |
| 12 | ACTN4 | Alpha-actinin-4 | 0.283 | 1.446 |
| 13 | ACTR3 | Actin-related protein 3 | 0.169 | 1.034 |
| 14 | ADAMTS13 | Isoform 2 of A disintegrin and metalloproteinase with thrombospondin motifs 13 | 0.589 | 0.477 |
| 15 | AFM | Afamin | 1.568 | 1.122 |
| 16 | AGMAT | Agmatinase, mitochondrial | 0.234 | 0.728 |
| 17 | AHCY | Adenosylhomocysteinase | — | 0.613 |
| 18 | AIFM1 | Isoform 3 of Apoptosis-inducing factor 1, mitochondrial | 0.121 | 1.011 |
| 19 | AK2 | Adenylate kinase 2, isoform CRA_a | 0.134 | 0.962 |
| 20 | AKR1A1 | Alcohol dehydrogenase [NADP(+)] | 0.074 | 0.812 |
| 21 | AKR1C1 | Aldo-keto reductase family 1 member C1 (Fragment) | 0.078 | 0.749 |
| 22 | ALBU | Serum albumin | 1.998 | 0.953 |
| 23 | ALDH1B1 | Aldehyde dehydrogenase X, mitochondrial | 0.221 | 1.028 |
| 24 | ALDH6A1 | Methylmalonate-semialdehyde dehydrogenase [acylating], mitochondrial | 0.113 | 0.661 |
| 25 | ALDH7A1 | Alpha-aminoadipic semialdehyde dehydrogenase | 0.129 | 0.973 |
| 26 | ALDH8A1 | Isoform 4 of Aldehyde dehydrogenase family 8 member A1 | 0.042 | — |
| 27 | ALDOA | Fructose-bisphosphate aldolase | 0.549 | 1.326 |
| 28 | ALYREF | THO complex subunit 4 | 0.323 | 1.244 |
| 29 | AMBP | Protein AMBP | — | 0.633 |
| 30 | AMY2B | Alpha-amylase 2B | 0.238 | 1.065 |
| 31 | ANXA5 | Annexin | 0.118 | 0.883 |
| 32 | ANXA6 | Annexin | 0.088 | — |
| 33 | AP1B1 | Isoform B of AP-1 complex subunit beta-1 | 0.098 | 0.698 |
| 34 | AP2A2 | Isoform 3 of AP-2 complex subunit alpha-2 | 0.183 | — |
| 35 | APCS | Serum amyloid P-component | 3.683 | 2.093 |
| 36 | APOA5 | Apolipoprotein A-V variant 3 | 1.613 | 1.481 |
| 37 | APOE | Apolipoprotein E (Fragment) | 1.56 | 1.125 |
| 38 | ARF3 | ADP-ribosylation factor 3 | 0.153 | 0.942 |
| 39 | ARHGAP1 | Rho GTPase-activating protein 1 | 0.556 | 0.805 |
| 40 | ARL1 | ADP-ribosylation factor-like protein 1 | 0.067 | 1.224 |
| 41 | ARPC2 | Actin-related protein 2/3 complex subunit 2 | 0.262 | 0.996 |
| 42 | ASL | Argininosuccinate lyase | 0.182 | 0.881 |
| 43 | ATIC | Homo sapiens 5-aminoimidazole-4-carboxamide ribonucleotide formyltransferase/IMP cyclohydrolase | 0.609 | 1.042 |
| 44 | ATL2 | Atlastin-2 | 0.234 | 0.924 |
| 45 | ATP5H | Isoform 2 of ATP synthase subunit d, mitochondrial | 0.238 | 1.075 |
| 46 | ATP6V1B2 | V-type proton ATPase subunit B, brain isoform (Fragment) | 0.290 | 1.244 |
| 47 | B4DPQ0 | Complement C1r subcomponent | — | 0.237 |
| 48 | BBOX1 | Homo sapiens butyrobetaine (gamma), 2-oxoglutarate dioxygenase (gamma-butyrobetaine hydroxylase) 1 | 0.278 | 1.023 |
| 49 | BHMT | betaine-homocysteine methyltransferase | — | 0.499 |
| 50 | BRL | Benzodiazepine receptor ligand | 0.085 | 0.512 |
| 51 | BRP44 | brain protein 44 | — | 0.381 |
| 52 | C1QB | Complement C1q subcomponent subunit B | — | 0.511 |
| 53 | C1QBP | complement component 1, q subcomponent binding protein | — | 1.572 |
| 54 | C1QC | Complement C1q subcomponent subunit C | — | 0.334 |
| 55 | C1RL | Complement C1r subcomponent-like protein | 0.313 | 0.209 |
| 56 | C4B | C4B (Fragment) | 1.964 | 2.097 |
| 57 | C4B | Complement C4B1a (Fragment) | 1.617 | 1.527 |
| 58 | C5 | Complement C5 | 2.849 | — |
| 59 | C6 | Complement component C6 | 3.158 | 2.953 |
| 60 | C7 | complement component 7 | 3.522 | — |
| 61 | C8A | Complement component C8 alpha chain | 2.480 | 1.463 |
| 62 | C8B | Complement component C8 beta chain | 2.062 | 1.396 |
| 63 | C8G | Complement component C8 gamma chain | 3.194 | 2.025 |
| 64 | CALM2 | Calmodulin (Fragment) | 0.318 | 1.001 |
| 65 | CAND1 | cullin-associated and neddylation-dissociated 1 | 0.247 | 0.873 |
| 66 | CARHSP1 | Calcium-regulated heat stable protein 1 | 0.099 | 0.820 |
| 67 | CBX3 | Chromobox protein homolog 3 | 0.419 | 1.025 |
| 68 | CCDC47 | Isoform 2 of Coiled-coil domain-containing protein 47 | 0.239 | 0.839 |
| 69 | CCT4 | T-complex protein 1 subunit delta | 0.216 | 0.889 |
| 70 | CCT6A | Homo sapiens chaperonin containing TCP1, subunit 6A (zeta 1) | 0.254 | 0.835 |
| 71 | CD5L | CD5 antigen-like | 1.747 | 1.024 |
| 72 | CDC123 | Cell division cycle protein 123 homolog | 0.024 | — |
| 73 | CDH1 | Truncated E-cadherin | 0.386 | 0.315 |
| 74 | CDH5 | Cadherin-5 | 0.645 | — |
| 75 | CENPV | Isoform 3 of Centromere protein V | 0.194 | 0.987 |
| 76 | CFH | complement factor H | 1.901 | 1.265 |
| 77 | CFH | Complement factor H CFH | 2.300 | 1.263 |
| 78 | CFHR1 | Complement factor H-related protein 1 | 2.579 | 1.587 |
| 79 | CFHR2 | Complement factor H-related protein 2 | 3.251 | 2.755 |
| 80 | CFP | Complement factor properdin, isoform CRA_c | 2.078 | — |
| 81 | CHDH | Choline dehydrogenase, mitochondrial | 0.119 | 0.627 |
| 82 | CHGA | Chromogranin-A | 1.971 | 1.442 |
| 83 | CISD1 | CDGSH iron-sulfur domain-containing protein 1 | 0.306 | 0.927 |
| 84 | CLTA | Isoform Non-brain of Clathrin light chain A | 0.101 | 0.893 |
| 85 | CLUH | Clustered mitochondria protein homolog (Fragment) | 0.205 | — |
| 86 | COMP | Cartilage oligomeric matrix protein | — | 0.640 |
| 87 | COMT | Catechol O-methyltransferase (Fragment) | 0.153 | 0.811 |
| 88 | COPA | Isoform 2 of Coatomer subunit alpha | 0.208 | 1.07 |
| 89 | COPB1 | Coatomer subunit beta | 0.182 | 0.768 |
| 90 | CP | ceruloplasmin (ferroxidase) | — | 0.344 |
| 91 | CPB2 | Carboxypeptidase B2 | 0.831 | 0.464 |
| 92 | CPN1 | Carboxypeptidase N catalytic chain | 1.902 | 1.441 |
| 93 | CPN2 | Carboxypeptidase N subunit 2 | 1.893 | 1.346 |
| 94 | CPNE3 | Copine-3 | 0.211 | 1.070 |
| 95 | CPOX | Coproporphyrinogen-III oxidase, mitochondrial | 0.054 | 0.717 |
| 96 | CRP | C-reactive protein | 2.354 | 0.614 |
| 97 | CRYZ | Quinone oxidoreductase (Fragment) | 0.284 | 1.113 |
| 98 | CSDE1 | Cold shock domain containing E1, RNA-binding, isoform CRA_d | 0.518 | 0.973 |
| 99 | CST3 | Cystatin-C | 1.525 | 1.046 |
| 100 | CYCS | Cytochrome c (Fragment) | — | 0.574 |
| 101 | CYP4A11 | Cytochrome P450 4A11 | 0.240 | 0.672 |
| 102 | CYP4Z1 | Cytochrome P450 4Z1 | 0.110 | 0.381 |
| 103 | DAK | FAD-AMP lyase (cyclizing) (Fragment) | 0.142 | 0.684 |
| 104 | DCTN2 | Dynactin subunit 2 (Fragment) | 0.443 | 0.878 |
| 105 | DDAH1 | N(G),N(G)-dimethylarginine dimethylaminohydrolase 1 | 0.202 | 0.955 |
| 106 | DDOST | Dolichyl-diphosphooligosaccharide--protein glycosyltransferase 48 kDa subunit | 0.212 | 0.895 |
| 107 | DDX1 | ATP-dependent RNA helicase DDX1 | 0.249 | 1.017 |
| 108 | DDX18 | Putative uncharacterized protein DDX18 (Fragment) | 0.535 | 1.075 |
| 109 | DDX5 | Probable ATP-dependent RNA helicase DDX5 | 0.373 | 1.025 |
| 110 | DHRS4L1 | Putative dehydrogenase/reductase SDR family member 4-like 2 | 0.135 | 0.787 |
| 111 | DKFZp686G11190 | Putative uncharacterized protein DKFZp686G11190 | 1.990 | 1.392 |
| 112 | DLST | DLST protein | 0.110 | 0.723 |
| 113 | DNAJC3 | DnaJ homolog subfamily C member 3 | 0.093 | — |
| 114 | DPP4 | Dipeptidyl peptidase 4 | 0.189 | 0.851 |
| 115 | DPYD | Dihydropyrimidine dehydrogenase [NADP(+)] | 0.093 | 0.981 |
| 116 | DSG2 | Desmoglein-2 | 0.605 | 0.540 |
| 117 | ECHDC1 | Ethylmalonyl-CoA decarboxylase (Fragment) | 0.291 | 0.969 |
| 118 | ECHS1 | Enoyl-CoA hydratase, mitochondrial | 0.186 | 0.781 |
| 119 | ECM1 | Truncated extracellular matrix protein 1 | — | 1.837 |
| 120 | EEF2 | Elongation factor 2 | 0.186 | 0.872 |
| 121 | EIF3A | Eukaryotic translation initiation factor 3 subunit A | 0.309 | 0.926 |
| 122 | EIF3B | Eukaryotic translation initiation factor 3 subunit B | 0.353 | 1.003 |
| 123 | EIF4A1 | eukaryotic translation initiation factor 4A, isoform 1 | 0.218 | 0.710 |
| 124 | ELAVL1 | ELAV-like protein 1 | 0.461 | 0.934 |
| 125 | ENO3 | Isoform 2 of Beta-enolase | 0.129 | 0.856 |
| 126 | EPFP1 | Chaperonin 10-related protein (Fragment) | 0.204 | 1.197 |
| 127 | ESD | S-formylglutathione hydrolase | 0.045 | — |
| 128 | ETFA | Electron transfer flavoprotein subunit alpha, mitochondrial | 0.068 | 0.806 |
| 129 | ETFDH | Electron transfer flavoprotein-ubiquinone oxidoreductase, mitochondrial | 0.236 | 0.674 |
| 130 | F10 | Coagulation factor X | 1.59 | 1.143 |
| 131 | F9p22 | Coagulation factor IX | 1.504 | 1.066 |
| 132 | FASN | Fatty acid synthase | 0.152 | 0.905 |
| 133 | FBLN1 | Isoform B of Fibulin-1 | 1.581 | 1.178 |
| 134 | FBP1 | fructose-1,6-bisphosphatase 1 | 0.063 | 0.401 |
| 135 | FES | Tyrosine-protein kinase Fes/Fps (Fragment) | 0.037 | — |
| 136 | FKBP2 | Peptidyl-prolyl cis-trans isomerase FKBP2 | 0.156 | 1.021 |
| 137 | FLNB | Isoform 2 of Filamin-B | 0.476 | 1.029 |
| 138 | G3BP | G3BP protein | 0.139 | 0.763 |
| 139 | GALM | Aldose 1-epimerase (Fragment) | 0.106 | 0.394 |
| 140 | GC | Vitamin D-binding protein | 1.596 | 1.293 |
| 141 | GCLC | Glutamate-cysteine ligase delta4 alternative splicing variant | 0.084 | 0.689 |
| 142 | GIG18 | Aspartate aminotransferase | 0.078 | 0.719 |
| 143 | GK | Isoform 1 of Glycerol kinase | 0.180 | 0.911 |
| 144 | GLO1 | Isoform 2 of Lactoylglutathione lyase | 0.082 | 0.844 |
| 145 | GLRX3 | Glutaredoxin-3 | 0.156 | 1.138 |
| 146 | GNB2 | Guanine nucleotide-binding protein G(I)/G(S)/G(T) subunit beta-2 | 0.187 | 1.120 |
| 147 | GPD1 | glycerol-3-phosphate dehydrogenase 1 | 0.084 | 0.875 |
| 148 | GPLD1 | Phosphatidylinositol-glycan-specific phospholipase D | 0.407 | 0.310 |
| 149 | GPT2 | Alanine aminotransferase 2 | 0.116 | 0.934 |
| 150 | GVINP1 | Interferon-induced very large GTPase 1 | — | 2.111 |
| 151 | HACL1 | 2-hydroxyacyl-CoA lyase 1 | — | 0.584 |
| 152 | HAGH | Hydroxyacylglutathione hydrolase, mitochondrial (Fragment) | 0.061 | — |
| 153 | HAL | Histidine ammonia-lyase | 0.116 | 0.684 |
| 154 | HBA1 | Hemoglobin alpha 1 | — | 0.544 |
| 155 | HBB | Hemoglobin subunit beta | — | 0.402 |
| 156 | HDLBP | high density lipoprotein binding protein | 0.232 | 0.872 |
| 157 | HGFAC | Hepatocyte growth factor activator | — | 0.659 |
| 158 | HIBADH | 3-hydroxyisobutyrate dehydrogenase, mitochondrial | 0.094 | 0.764 |
| 159 | HIST1H1E | Homo sapiens histone 1, H1e | 0.185 | 1.366 |
| 160 | HMGB1 | High mobility group protein B1 (Fragment) | 0.156 | 1.424 |
| 161 | HMGCS2 | Isoform 2 of Hydroxymethylglutaryl-CoA synthase, mitochondrial | — | 0.605 |
| 162 | HNRNPA0 | Heterogeneous nuclear ribonucleoprotein A0 | 0.505 | 0.972 |
| 163 | HNRNPD | Heterogeneous nuclear ribonucleoprotein D0 (Fragment) | 0.317 | 1.036 |
| 164 | HNRNPH1 | Heterogeneous nuclear ribonucleoprotein H | 0.429 | 1.142 |
| 165 | HNRPAB | heterogeneous nuclear ribonucleoprotein A/B | 0.057 | 1.067 |
| 166 | HPR | Isoform 2 of Haptoglobin-related protein | 1.513 | 1.094 |
| 167 | HPX | Hemopexin | 0.787 | 0.580 |
| 168 | HSD11B1 | Corticosteroid 11-beta-dehydrogenase isozyme 1 | 0.154 | 0.449 |
| 169 | HSD17B11 | Estradiol 17-beta-dehydrogenase 11 | 0.141 | 0.555 |
| 170 | HSD17B4 | hydroxysteroid (17-beta) dehydrogenase 4 | 0.127 | 0.767 |
| 171 | HSP90AA1 | Isoform 2 of Heat shock protein HSP 90-alpha | 0.236 | 0.938 |
| 172 | HSPA5 | 78 kDa glucose-regulated protein | 0.284 | 0.986 |
| 173 | IARS2 | Isoleucine--tRNA ligase, mitochondrial | 1.127 | 1.758 |
| 174 | IDH1 | Isocitrate dehydrogenase [NADP] | 0.074 | 0.864 |
| 175 | IDH2 | Isocitrate dehydrogenase [NADP] | 0.078 | 0.977 |
| 176 | IDH3A | Isocitrate dehydrogenase [NAD] subunit alpha, mitochondrial | 0.132 | 0.917 |
| 177 | IDH3B | Isoform A of Isocitrate dehydrogenase [NAD] subunit beta, mitochondrial | 0.214 | 0.974 |
| 178 | IGJ | Immunoglobulin J chain | — | 2.238 |
| 179 | INHBC | cDNA, FLJ95746, highly similar to Homo sapiens inhibin, beta C (INHBC), mRNA PE=2 SV=1 | 1.598 | 1.012 |
| 180 | ITGB1 | Integrin beta-1 | 0.505 | 1.041 |
| 181 | ITIH3 | Isoform 2 of Inter-alpha-trypsin inhibitor heavy chain H3 | 1.546 | 1.139 |
| 182 | IVD | Isovaleryl Coenzyme A dehydrogenase, isoform CRA_a | 0.094 | 0.689 |
| 183 | JUP | Junction plakoglobin | 0.336 | 1.174 |
| 184 | KHK | Isoform C of Ketohexokinase | 0.167 | 0.967 |
| 185 | KLKB1 | Plasma kallikrein heavy chain (Fragment) | 3.402 | — |
| 186 | KNG1 | Kininogen 1, isoform CRA_b | — | 2.007 |
| 187 | KPNA3 | Importin subunit alpha | 0.204 | 0.938 |
| 188 | KPNB1 | karyopherin (importin) beta 1 | 0.347 | 0.943 |
| 189 | KRT10 | Keratin, type I cytoskeletal 10 | 0.909 | 0.637 |
| 190 | KRT2 | Keratin, type II cytoskeletal 2 epidermal | — | 0.483 |
| 191 | LAP3 | Isoform 2 of Cytosol aminopeptidase | 0.097 | 0.781 |
| 192 | LGALS8 | Galectin (Fragment) | 0.386 | 1.121 |
| 193 | LIMA1 | Isoform 4 of LIM domain and actin-binding protein 1 | 0.104 | 0.840 |
| 194 | LMAN1 | lectin, mannose-binding, 1 | 0.566 | 1.105 |
| 195 | LUM | Lumican | 1.854 | 1.279 |
| 196 | MASP1 | Isoform 2 of Mannan-binding lectin serine protease 1 | — | 0.651 |
| 197 | MASP1 | Mannan-binding lectin serine protease 1 | 0.600 | 0.419 |
| 198 | MDH2 | Malate dehydrogenase (Fragment) | 0.091 | 1.158 |
| 199 | MMP2 | 72 kDa type IV collagenase | 0.316 | — |
| 200 | MSN | MSN protein (Fragment) | 0.224 | 0.950 |
| 201 | MTHFD1 | C-1-tetrahydrofolate synthase, cytoplasmic | 0.227 | 1.064 |
| 202 | MYO1B | Unconventional myosin-Ib | 0.425 | 0.934 |
| 203 | NACA | Nascent polypeptide-associated complex subunit alpha (Fragment) | 0.079 | 0.881 |
| 204 | NDUFA4 | NADH dehydrogenase [ubiquinone] 1 alpha subcomplex subunit 4 | 0.072 | 0.656 |
| 205 | NDUFAB1 | Acyl carrier protein, mitochondrial | 0.174 | 0.868 |
| 206 | NDUFS1 | Isoform 2 of NADH-ubiquinone oxidoreductase 75 kDa subunit, mitochondrial | 0.258 | 0.983 |
| 207 | NDUFV2 | NDUFV2 protein | 0.183 | 1.016 |
| 208 | NIPSNAP1 | nipsnap homolog 1 | 0.238 | 0.932 |
| 209 | NIT2 | Omega-amidase NIT2 | 0.048 | 0.695 |
| 210 | NME1-NME2 | Nucleoside diphosphate kinase | 0.182 | 0.814 |
| 211 | NOMO3 | Nodal modulator 3 | 0.341 | 0.995 |
| 212 | NONO | non-POU domain containing, octamer-binding | 0.280 | 0.878 |
| 213 | NUCB1 | nucleobindin 1 | 0.573 | 0.925 |
| 214 | NUDT4 | nudix (nucleoside diphosphate linked moiety X)-typemotif 4 | — | 0.529 |
| 215 | OAF | Out at first protein homolog | 1.62 | 1.157 |
| 216 | OAT | Ornithine aminotransferase, mitochondrial | 0.115 | 0.818 |
| 217 | OGDH | 2-oxoglutarate dehydrogenase, mitochondrial | 0.100 | — |
| 218 | OPA1 | Dynamin-like 120 kDa protein, mitochondrial | 0.146 | 1.075 |
| 219 | PA2G4 | PA2G4 protein (Fragment) | 0.391 | 1.011 |
| 220 | PACSIN3 | Protein kinase C and casein kinase substrate in neurons 3, isoform CRA_b | 0.198 | 1.138 |
| 221 | PAH | Homo sapiens phenylalanine hydroxylase (PAH) | 0.073 | 0.739 |
| 222 | PCBP1 | Poly(rC)-binding protein 1 | 0.231 | 0.921 |
| 223 | PCNA | Proliferating cell nuclear antigen (Fragment) | 0.539 | 1.145 |
| 224 | PDHA1 | Pyruvate dehydrogenase E1 component subunit alpha, somatic form, mitochondrial | 0.205 | — |
| 225 | PDIA4 | Protein disulfide-isomerase A4 | 0.315 | 0.942 |
| 226 | PDLIM1 | PDZ and LIM domain protein 1 | 0.191 | 1.184 |
| 227 | PF4 | Platelet factor 4 | 0.449 | 0.432 |
| 228 | PGAM1 | Phosphoglycerate mutase 1 (Brain) | 0.251 | 0.898 |
| 229 | PGK1 | Phosphoglycerate kinase | 0.142 | 1.026 |
| 230 | PGLYRP | peptidoglycan recognition protein L | — | 0.655 |
| 231 | PGRMC1 | Membrane-associated progesterone receptor component 1 | 0.166 | 0.803 |
| 232 | PLS3 | Plastin-3 | 0.180 | 1.078 |
| 233 | PON3 | Serum paraoxonase/lactonase 3 | — | 0.524 |
| 234 | PRDX1 | peroxiredoxin 1 | 0.073 | 0.710 |
| 235 | PRDX6 | Peroxiredoxin-6 | 0.294 | 0.580 |
| 236 | PSMA1 | Isoform Long of Proteasome subunit alpha type-1 | 0.364 | 0.908 |
| 237 | PSMA3 | Proteasome subunit alpha type | 0.286 | 0.819 |
| 238 | PSMA6 | Proteasome subunit alpha type | 0.218 | 0.807 |
| 239 | PSMA7 | Proteasome subunit alpha type (Fragment) | 0.276 | 0.790 |
| 240 | PSMC3 | 26S protease regulatory subunit 6A GN=PSMC3 PE=4 SV=1 | 0.242 | 0.825 |
| 241 | PSMC4 | Isoform 2 of 26S protease regulatory subunit 6B | 0.249 | — |
| 242 | PSMC5 | 26S protease regulatory subunit 8 (Fragment) | 0.337 | 0.738 |
| 243 | PSMD1 | proteasome (prosome, macropain) 26S subunit, non-ATPase, 1 | 0.179 | 0.865 |
| 244 | PSMD12 | Isoform 2 of 26S proteasome non-ATPase regulatory subunit 12 | 0.128 | 0.494 |
| 245 | PSMD7 | proteasome (prosome, macropain) 26S subunit, non-ATPase, 7 (Mov34 homolog) | 0.181 | 0.827 |
| 246 | Q86TT1 | Full-length cDNA clone CS0DD006YL02 of Neuroblastoma of Homo sapiens (human) | 1.721 | 1.114 |
| 247 | QDPR | Dihydropteridine reductase | 0.116 | 0.667 |
| 248 | RAB11A | Ras-related protein Rab-11A (Fragment) | 0.178 | 0.875 |
| 249 | RAB14 | Ras-related protein Rab-14 | 0.341 | 1.079 |
| 250 | RAB1A | Ras-related protein Rab-1A | 0.338 | 0.945 |
| 251 | RAB2A | Ras-related protein Rab-2A | 0.567 | 0.987 |
| 252 | Raichu404X | Raichu404X | 0.468 | 1.136 |
| 253 | RAN | GTP-binding nuclear protein Ran (Fragment) | 0.111 | 0.782 |
| 254 | RBMX | RNA-binding motif protein, X chromosome, N-terminally processed | 0.245 | 0.930 |
| 255 | RBP4 | Plasma retinol-binding protein(1-182) | 3.155 | 1.271 |
| 256 | RDX | Isoform 5 of Radixin | 0.117 | 0.623 |
| 257 | RELN | Reelin | — | 0.558 |
| 258 | RGN | Regucalcin | 0.103 | 0.556 |
| 259 | RPL10A | Ribosomal protein (Fragment) | 0.293 | 1.086 |
| 260 | RPL14 | ribosomal protein L14 | 0.081 | 0.808 |
| 261 | RPL15 | Ribosomal protein L15 (Fragment) GN=RPL15 PE=3 SV=1 | 0.084 | 0.529 |
| 262 | RPL18A | 60S ribosomal protein L18a | 0.038 | 1.040 |
| 263 | RPL24 | 60S ribosomal protein L24 | 0.106 | 0.977 |
| 264 | RPL27 | 60S ribosomal protein L27 PE=2 SV=1 | 0.059 | 0.754 |
| 265 | RPL3 | 60S ribosomal protein L3 (Fragment) | 0.346 | 0.955 |
| 266 | RPL30 | 60S ribosomal protein L30 (Fragment) | 0.119 | 0.911 |
| 267 | RPL32 | 60S ribosomal protein L32 (Fragment) | 0.123 | 0.797 |
| 268 | RPL4 | 60S ribosomal protein L4 | 0.087 | 0.772 |
| 269 | RPL5 | Ribosomal protein L5, isoform CRA_b | 0.291 | 0.923 |
| 270 | RPL7A | 60S ribosomal protein L7a (Fragment) | 0.083 | — |
| 271 | RPL9 | 60S ribosomal protein L9 (Fragment) | 0.096 | 0.708 |
| 272 | RPN1 | RPN1 protein | 0.252 | 1.052 |
| 273 | RPS10 | 40S ribosomal protein S10 | 0.042 | 1.003 |
| 274 | RPS14 | 40S ribosomal protein S14 | 0.298 | 1.069 |
| 275 | RPS16 | 40S ribosomal protein S16 | 0.129 | 0.879 |
| 276 | RPS17 | 40S ribosomal protein S17 | 0.233 | 0.778 |
| 277 | RPS2 | 40S ribosomal protein S2 (Fragment) | 0.129 | 0.625 |
| 278 | RPS23 | 40S ribosomal protein S23 | 0.074 | 0.908 |
| 279 | RPS24 | 40S ribosomal protein S24 | 0.093 | 0.572 |
| 280 | RPS25 | 40S ribosomal protein S25 | 0.095 | 1.015 |
| 281 | RPS26 | HCG1745083 | 0.070 | 1.026 |
| 282 | RPS27 | 40S ribosomal protein S27 | 0.358 | 1.217 |
| 283 | RPS27A | Ribosomal protein S27a | 0.224 | 0.858 |
| 284 | RPS3A | 40S ribosomal protein S3a (Fragment) | 0.185 | 0.803 |
| 285 | RPS8 | 40S ribosomal protein S8 | 0.111 | 0.734 |
| 286 | RPS9 | 40S ribosomal protein S9 | 0.038 | 0.331 |
| 287 | RPSA | 40S ribosomal protein SA (Fragment) | 0.157 | 0.956 |
| 288 | SAA2 | Serum amyloid A-2 protein | — | 0.647 |
| 289 | SAP18 | Histone deacetylase complex subunit SAP18 (Fragment) | 0.622 | 0.869 |
| 290 | SARDH | Sarcosine dehydrogenase, mitochondrial | 0.241 | 0.873 |
| 291 | SCP2 | sterol carrier protein 2 | 0.104 | 0.917 |
| 292 | SDHA | Succinate dehydrogenase [ubiquinone] flavoprotein subunit, mitochondrial | 0.192 | 1.004 |
| 293 | SDHB | Succinate dehydrogenase complex subunit B (Fragment) | 0.146 | 0.927 |
| 294 | SEC13 | Protein SEC13 homolog | 0.278 | 0.898 |
| 295 | SEC14L4 | SEC14-like protein 4 | 0.130 | 1.081 |
| 296 | SELENBP1 | Selenium-binding protein 1 | 0.087 | 0.770 |
| 297 | SEPP1 | Selenoprotein P (Fragment) | 2.639 | 2.071 |
| 298 | SEPT2 | Septin-2 | 0.262 | 0.849 |
| 299 | SEPT7 | Septin-7 (Fragment) | 0.277 | 1.006 |
| 300 | SERPINA10 | Protein Z-dependent protease inhibitor | 1.651 | 0.878 |
| 301 | SERPINA6 | Homo sapiens serine (or cysteine) proteinase inhibitor, clade A(alpha-1 antiproteinase, antitrypsin), member 6 | 1.517 | 1.006 |
| 302 | SERPINB8 | Homo sapiens serine (or cysteine) proteinase inhibitor, clade B (ovalbumin), member 8 | 0.107 | 0.983 |
| 303 | SET | SET translocation (myeloid leukemia-associated) | 0.209 | 1.343 |
| 304 | SF3B3 | splicing factor 3b, subunit 3, | 0.338 | 0.834 |
| 305 | SHMT1 | Serine hydroxymethyltransferase | 0.16 | 0.714 |
| 306 | SLC25A13 | Isoform 2 of Calcium-binding mitochondrial carrier protein Aralar2 | 0.114 | 0.982 |
| 307 | SLC25A15 | Homo sapiens solute carrier family 25member 15, nuclear gene encoding mitochondrial protein | 0.261 | 0.978 |
| 308 | SLC25A3 | solute carrier family 25 (mitochondrial carrier;phosphate carrier), member 3 (SLC25A3), nuclear gene encodingmitochondrial protein, transcript variant 1b | 0.121 | 0.77 |
| 309 | SND1 | staphylococcal nuclease domain containing 1 | 0.150 | 0.760 |
| 310 | SNRNP200 | U5 small nuclear ribonucleoprotein 200 kDa helicase | 0.262 | 0.904 |
| 311 | SNRPD3 | Small nuclear ribonucleoprotein Sm D3 | 0.228 | 0.853 |
| 312 | SNRPE | Small nuclear ribonucleoprotein E | 0.207 | 0.862 |
| 313 | SNRPG | Small nuclear ribonucleoprotein G | 0.135 | 0.922 |
| 314 | sp\|P01611\|KV119 | Ig kappa chain V-I region Wes | 1.563 | 0.944 |
| 315 | sp\|P01613\|KV121 | Ig kappa chain V-I region Ni | 1.59 | 1.025 |
| 316 | sp\|P01625\|KV402 | Ig kappa chain V-IV region Len | 1.731 | 0.876 |
| 317 | sp\|P01714\|LV301 | Ig lambda chain V-III region SH | 1.559 | 0.669 |
| 318 | sp\|P04430\|KV122 | Ig kappa chain V-I region BAN | 1.628 | 1.007 |
| 319 | sp\|P06311\|KV311 | Ig kappa chain V-III region IARC/BL41 | 1.565 | 0.855 |
| 320 | SPARC | Secreted protein, acidic, cysteine-rich (Osteonectin), isoform CRA_a | 0.394 | — |
| 321 | SPTAN1 | Spectrin alpha chain, non-erythrocytic 1 | 0.280 | 1.138 |
| 322 | SQRDL | sulfide quinone reductase-like | 0.655 | 1.023 |
| 323 | SRSF1 | Serine/arginine-rich-splicing factor 1 (Fragment) | 0.174 | 1.055 |
| 324 | SSR1 | signal sequence receptor, alpha (translocon-associated protein alpha) | 0.268 | 1.258 |
| 325 | SSR3 | Translocon-associated protein subunit gamma | 0.225 | 0.897 |
| 326 | ST13 | ST13 protein (Fragment) | 0.198 | 0.908 |
| 327 | ST1A5 | Phenol sulfotransferase 1A5*5 | 0.154 | 0.595 |
| 328 | STARD5 | StAR-related lipid transfer protein 5 | 0.035 | 0.718 |
| 329 | STT3A | Dolichyl-diphosphooligosaccharide--protein glycosyltransferase subunit STT3A | 0.164 | 0.994 |
| 330 | SUCLA2 | cDNA FLJ53646, highly similar to Succinyl-CoA ligase (ADP-forming) beta-chain, mitochondrial | 0.130 | 0.669 |
| 331 | SUCLG1 | Homo sapiens succinate-CoA ligase, GDP-forming, alpha subunit | 0.122 | 1.032 |
| 332 | SUCLG2 | SUCLG2 protein (Fragment) | 0.076 | 0.631 |
| 333 | SYNCRIP | SYNCRIP protein (Fragment) | 0.438 | 0.928 |
| 334 | TALDO1 | Transaldolase | 0.163 | 0.841 |
| 335 | TCEB1 | Transcription elongation factor B polypeptide 1 (Fragment) | 0.383 | 1.149 |
| 336 | TCP1 | T-complex protein 1 subunit alpha | 0.244 | 0.918 |
| 337 | TFRC | Transferrin receptor (P90, CD71), isoform CRA_c | 0.628 | 0.749 |
| 338 | TIMM13 | Mitochondrial import inner membrane translocase subunit Tim13 | 0.191 | 1.039 |
| 339 | TKT | Transketolase | 0.101 | 0.900 |
| 340 | TLN1 | Talin-1 | 0.785 | 1.830 |
| 341 | TMPO | Lamina-associated polypeptide 2, isoform alpha | 0.496 | 0.899 |
| 342 | TOP1 | Topoisomerase (DNA) I | 0.193 | 0.990 |
| 343 | TPM3-ROS1 | Tyrosine-protein kinase receptor | 0.185 | 1.019 |
| 344 | tr\|A0A5E4\|A0A5E4 | Uncharacterized protein | 1.993 | 0.956 |
| 345 | tr\|A2N011\|A2N011 | Vh1-D-J3-region (Fragment) | 1.717 | 1.200 |
| 346 | tr\|A8K2T4\|A8K2T4 | cDNA FLJ78207, highly similar to Human complement protein component C7 mRNA | — | 3.924 |
| 347 | tr\|B0AZL7\|B0AZL7 | cDNA, FLJ79457, highly similar to Insulin-like growth factor-binding proteincomplex acid labile chain | 1.627 | 1.292 |
| 348 | tr\|B2R4A2\|B2R4A2 | Cytochrome b-c1 complex subunit 7 | 0.307 | 0.886 |
| 349 | tr\|B2R920\|B2R920 | Glutathione S-transferase | — | 0.526 |
| 350 | tr\|B2RCG5\|B2RCG5 | Dimethylaniline monooxygenase [N-oxide-forming] | 0.208 | 0.788 |
| 351 | tr\|B3KM97\|B3KM97 | cDNA FLJ10554 fis, clone NT2RP2002385, highly similar to Synaptic glycoprotein SC2 | 0.075 | 0.748 |
| 352 | tr\|B3KMI9\|B3KMI9 | cDNA FLJ11140 fis, clone PLACE1006488, highly similar to Signal recognition particle 68 kDa protein | 0.329 | 0.876 |
| 353 | tr\|B3KNX0\|B3KNX0 | cDNA FLJ30621 fis, clone CTONG2001681, highly similar to Complement C1s subcomponent (EC 3.4.21.42) | — | 0.208 |
| 354 | tr\|B3KQ51\|B3KQ51 | Serine/threonine-protein phosphatase PE=2 SV=1 | 0.250 | 0.830 |
| 355 | tr\|B3KQ84\|B3KQ84 | cDNA FLJ33079 fis, clone TRACH2000306, highly similar to Dimethylglycine dehydrogenase, mitochondrial | 0.094 | 0.411 |
| 356 | tr\|B3KRN4\|B3KRN4 | cDNA FLJ34625 fis, clone KIDNE2015244, highly similar to Serine protease HTRA1 (EC 3.4.21.-) | 1.759 | 1.015 |
| 357 | tr\|B3KRV7\|B3KRV7 | cDNA FLJ34967 fis, clone NTONG2004690, highly similar to LIPOPROTEIN LIPASE (EC 3.1.1.34) | 1.845 | 1.200 |
| 358 | tr\|B3KS53\|B3KS53 | cDNA FLJ35535 fis, clone SPLEN2002419, highly similar to EGF-containing fibulin-like extracellular matrix protein 1 | 1.524 | 1.155 |
| 359 | tr\|B3KSG9\|B3KSG9 | cDNA FLJ36188 fis, clone TESTI2027179, highly similar to Transmembrane 9 superfamily protein member 2 | 0.403 | 0.972 |
| 360 | tr\|B3KT93\|B3KT93 | cDNA FLJ37875 fis, clone BRSSN2018771, highly similar to Poly(A)-binding protein 1 | 0.082 | 0.826 |
| 361 | tr\|B3KTJ1\|B3KTJ1 | cDNA FLJ38349 fis, clone FEBRA1000057, highly similar to PCTP-like protein | 0.131 | 0.610 |
| 362 | tr\|B3KTN2\|B3KTN2 | cDNA FLJ38533 fis, clone HCHON2001108, highly similar to Threonyl-tRNA synthetase, cytoplasmic | 0.344 | 0.984 |
| 363 | tr\|B3KUZ8\|B3KUZ8 | Aspartate aminotransferase | 0.127 | 0.685 |
| 364 | tr\|B3KWD9\|B3KWD9 | cDNA FLJ42834 fis, clone BRCAN2019002, highly similar to GTP:AMP phosphotransferase mitochondrial (EC 2.7.4.10) PE=2 SV=1 | 0.203 | 1.035 |
| 365 | tr\|B3KY43\|B3KY43 | cDNA FLJ46798 fis, clone TRACH3031660, highly similar to cAMP-dependent protein kinase type II-beta regulatory subunit | 0.198 | 0.902 |
| 366 | tr\|B3KY95\|B3KY95 | cDNA FLJ16143 fis, clone BRAMY2038516, highly similar to Protein disulfide-isomerase A6 | 0.154 | 1.084 |
| 367 | tr\|B3VL17\|B3VL17 | Beta globin (Fragment) | — | 0.439 |
| 368 | tr\|B4DDF7\|B4DDF7 | cDNA FLJ53296, highly similar to Serine/threonine-protein phosphatase 2A 65 kDa regulatory subunit A alpha isoform PE=2 SV=1 | 0.133 | 0.714 |
| 369 | tr\|B4DDF8\|B4DDF8 | cDNA FLJ51786, highly similar to Retinal dehydrogenase 1 | 0.116 | 0.695 |
| 370 | tr\|B4DE78\|B4DE78 | cDNA FLJ52141, highly similar to 14-3-3 protein gamma | 0.339 | 0.878 |
| 371 | tr\|B4DE91\|B4DE91 | cDNA FLJ55534, highly similar to 4-trimethylaminobutyraldehyde dehydrogenase | 0.053 | 0.872 |
| 372 | tr\|B4DEA8\|B4DEA8 | cDNA FLJ56425, highly similar to Very-long-chain specific acyl-CoAdehydrogenase, mitochondrial | 0.144 | 0.881 |
| 373 | tr\|B4DEN6\|B4DEN6 | cDNA FLJ55549, highly similar to 3-ketoacyl-CoA thiolase, peroxisomal | 0.082 | 0.795 |
| 374 | tr\|B4DF70\|B4DF70 | cDNA FLJ60461, highly similar to Peroxiredoxin-2 | 0.426 | 0.377 |
| 375 | tr\|B4DFE6\|B4DFE6 | cDNA FLJ59861, highly similar to ATP synthase gamma chain, mitochondrial | 0.110 | 0.591 |
| 376 | tr\|B4DFK9\|B4DFK9 | cDNA FLJ57964, highly similar to Heterogeneous nuclear ribonucleoprotein H~ | 0.436 | 1.053 |
| 377 | tr\|B4DFL1\|B4DFL1 | Dihydrolipoyl dehydrogenase | 0.393 | 0.982 |
| 378 | tr\|B4DFM1\|B4DFM1 | cDNA FLJ53034, highly similar to AP-2 complex subunit mu-1 | 0.363 | 1.011 |
| 379 | tr\|B4DGH9\|B4DGH9 | cDNA FLJ58772, highly similar to Fragile X mental retardation syndrome-related protein 1 | 0.492 | 0.895 |
| 380 | tr\|B4DH02\|B4DH02 | cDNA FLJ50510, highly similar to Heat shock 70 kDa protein 4 | 0.297 | 0.960 |
| 381 | tr\|B4DH43\|B4DH43 | cDNA FLJ56433, highly similar to Collagen alpha-2(IV) chain | 0.107 | 0.887 |
| 382 | tr\|B4DIC4\|B4DIC4 | cDNA FLJ52195, highly similar to LIM and SH3 domain protein | 0.221 | 0.937 |
| 383 | tr\|B4DJ30\|B4DJ30 | cDNA FLJ61290, highly similar to Neutral alpha-glucosidase AB | 0.518 | 1.141 |
| 384 | tr\|B4DKS8\|B4DKS8 | cDNA FLJ57121, highly similar to Heterogeneous nuclear ribonucleoprotein F | 0.475 | 0.983 |
| 385 | tr\|B4DL07\|B4DL07 | cDNA FLJ53353, highly similar to ATP-binding cassette sub-family D member 3 | — | 0.656 |
| 386 | tr\|B4DL49\|B4DL49 | cDNA FLJ58073, moderately similar to Cathepsin B (EC 3.4.22.1) | — | 0.320 |
| 387 | tr\|B4DLV7\|B4DLV7 | cDNA FLJ60299, highly similar to Rab GDP dissociation inhibitor beta | 0.239 | 0.982 |
| 388 | tr\|B4DM22\|B4DM22 | cDNA FLJ53357, highly similar to 26S proteasome non-ATPase regulatory subunit 2 | 0.170 | — |
| 389 | tr\|B4DNR3\|B4DNR3 | cDNA FLJ52710, highly similar to Abhydrolase domain-containing protein 14B | 0.002 | 0.281 |
| 390 | tr\|B4DP27\|B4DP27 | cDNA FLJ52153, highly similar to Transmembrane emp24 domain-containing protein 2 | 0.277 | 0.922 |
| 391 | tr\|B4DPJ2\|B4DPJ2 | Annexin | 0.603 | 0.927 |
| 392 | tr\|B4DPQ9\|B4DPQ9 | cDNA FLJ58095, highly similar to Cytochrome P450 2C9 | 0.054 | 0.208 |
| 393 | tr\|B4DRH6\|B4DRH6 | cDNA FLJ54509, highly similar to Trifunctional enzyme subunit alpha, mitochondrial | 0.478 | 0.933 |
| 394 | tr\|B4DRR0\|B4DRR0 | cDNA FLJ53910, highly similar to Keratin, type II cytoskeletal 6A | 0.447 | 0.915 |
| 395 | tr\|B4DRW3\|B4DRW3 | cDNA FLJ57180, highly similar to TAR DNA-binding protein 43 | 0.125 | 0.909 |
| 396 | tr\|B4DT97\|B4DT97 | cDNA FLJ56931, highly similar to Peroxisomal carnitine O-octanoyltransferase | 0.076 | --- |
| 397 | tr\|B4DTS5\|B4DTS5 | cDNA FLJ58882, highly similar to 26S proteasome non-ATPase regulatory subunit 11 | 0.198 | 0.845 |
| 398 | tr\|B4DU07\|B4DU07 | Acyl-coenzyme A oxidase | — | 0.519 |
| 399 | tr\|B4DU91\|B4DU91 | cDNA FLJ55368, highly similar to Epsin-1 | 0.121 | 0.928 |
| 400 | tr\|B4DUP2\|B4DUP2 | cDNA FLJ56155, highly similar to UTP--glucose-1-phosphate uridylyltransferase 2 | 0.249 | 0.805 |
| 401 | tr\|B4DUQ1\|B4DUQ1 | cDNA FLJ54552, highly similar to Heterogeneous nuclear ribonucleoprotein K | 0.151 | 1.062 |
| 402 | tr\|B4DUV1\|B4DUV1 | cDNA FLJ53207, highly similar to Homo sapiens fibulin 1 (FBLN1), transcript variant C, mRNA | 1.704 | 1.309 |
| 403 | tr\|B4DW81\|B4DW81 | cDNA FLJ58863, highly similar to Protein NipSnap3A | 0.352 | 0.880 |
| 404 | tr\|B4DZ87\|B4DZ87 | cDNA FLJ57240, highly similar to Mitochondrial proteins import receptor | 0.217 | 0.883 |
| 405 | tr\|B4DZP5\|B4DZP5 | cDNA FLJ51165, highly similar to DNA damage-binding protein 1 PE=2 SV=1 | 0.083 | 1.166 |
| 406 | tr\|B4E0S6\|B4E0S6 | cDNA FLJ55635, highly similar to pre-mRNA-splicing factorATP-dependent RNA helicase DHX15 | 0.345 | 0.794 |
| 407 | tr\|B4E1D0\|B4E1D0 | cDNA FLJ53951, highly similar to Glucokinase regulatory protein | 0.053 | — |
| 408 | tr\|B4E1E3\|B4E1E3 | cDNA FLJ61033, highly similar to APOBEC1 complementation factor | 0.190 | 1.323 |
| 409 | tr\|B4E1Q7\|B4E1Q7 | cDNA FLJ57294, highly similar to Lipoamide acyltransferase component of branched-chain alpha-keto acid dehydrogenase complex, mitochondrial | 0.074 | 0.799 |
| 410 | tr\|B4E2A6\|B4E2A6 | cDNA FLJ55508, highly similar to Sad1/unc-84-like protein 2 | 0.520 | 1.355 |
| 411 | tr\|B4E2G8\|B4E2G8 | cDNA FLJ54047, highly similar to Alpha-1 catenin (Cadherin-associated protein) | 0.614 | 1.214 |
| 412 | tr\|B5FX47\|B5FX47 | 4-hydroxyphenylpyruvate dioxygenase (Fragment) | 0.064 | 0.349 |
| 413 | tr\|B7Z4P9\|B7Z4P9 | cDNA FLJ51678, highly similar to Ras-related protein Rab-18 | 0.290 | 0.606 |
| 414 | tr\|B7Z4W4\|B7Z4W4 | cDNA FLJ50817, highly similar to UV excision repair protein RAD23 homolog B | 0.216 | 0.993 |
| 415 | tr\|B7Z539\|B7Z539 | cDNA FLJ56954, highly similar to Inter-alpha-trypsin inhibitor heavy chain H1 | — | 0.616 |
| 416 | tr\|B7Z565\|B7Z565 | cDNA FLJ54739, highly similar to Alpha-actinin-1 | 0.596 | 1.602 |
| 417 | tr\|B7Z601\|B7Z601 | cDNA FLJ57187, highly similar to Glycerol-3-phosphate dehydrogenase, mitochondrial | 0.387 | 1.118 |
| 418 | tr\|B7Z795\|B7Z795 | cDNA FLJ57637, highly similar to Liver carboxylesterase 1 | 0.193 | 0.949 |
| 419 | tr\|B7Z7U6\|B7Z7U6 | cDNA FLJ51769, highly similar to Ras GTPase-activating-like protein IQGAP2 (Fragment) | 0.219 | 0.781 |
| 420 | tr\|D3JV41\|D3JV41 | Thrombocidin-2 antimicrobial variant (Fragment) | 0.361 | 0.330 |
| 421 | tr\|E5KN59\|E5KN59 | Peptidyl-prolyl cis-trans isomerase D | 0.234 | 1.331 |
| 422 | tr\|F2YGG7\|F2YGG7 | Epidermal growth factor receptor | 0.471 | 1.337 |
| 423 | tr\|Q08ES8\|Q08ES8 | Cell growth-inhibiting protein 34 | 0.204 | 0.835 |
| 424 | tr\|Q2TU64\|Q2TU64 | PIG48 | 0.183 | 1.160 |
| 425 | tr\|Q3YA63\|Q3YA63 | Cytochrome P450 2E1 (Fragment) | 0.091 | 0.920 |
| 426 | tr\|Q53FT8\|Q53FT8 | Proteasome subunit beta type (Fragment) | 0.278 | 0.795 |
| 427 | tr\|Q53GS2\|Q53GS2 | Solute carrier family 27 (Fatty acid transporter), member 2 variant (Fragment) | 0.071 | — |
| 428 | tr\|Q53HU0\|Q53HU0 | Chaperonin containing TCP1, subunit 8 (Theta) variant (Fragment) | 0.240 | 0.907 |
| 429 | tr\|Q53HV2\|Q53HV2 | Chaperonin containing TCP1, subunit 7 (Eta) variant (Fragment) | 0.436 | 1.121 |
| 430 | tr\|Q59E99\|Q59E99 | Thrombospondin 1 variant (Fragment) | 0.549 | 0.583 |
| 431 | tr\|Q59EI9\|Q59EI9 | ADP,ATP carrier protein, liver isoform T2 variant (Fragment) | 0.392 | 0.983 |
| 432 | tr\|Q5U025\|Q5U025 | ADP-ribosylation factor 6 | 0.131 | 0.618 |
| 433 | tr\|Q6ZMU0\|Q6ZMU0 | Delta-aminolevulinic acid dehydratase | 0.101 | 0.638 |
| 434 | tr\|Q71V99\|Q71V99 | Peptidyl-prolyl cis-trans isomerase | 0.130 | 0.721 |
| 435 | tr\|Q92799\|Q92799 | Mitochondrial trifunctional protein beta subunit (Fragment) | 0.304 | 0.822 |
| 436 | tr\|Q96K68\|Q96K68 | cDNA FLJ14473 fis, clone MAMMA1001080, highly similar to Homo sapiens SNC73 protein (SNC73) mRNA | 1.681 | 1.147 |
| 437 | tr\|Q9BUM6\|Q9BUM6 | COL6A2 protein | 0.180 | 0.756 |
| 438 | tr\|Q9H552\|Q9H552 | Uncharacterized protein | 0.061 | 0.744 |
| 439 | tr\|Q9NPP6\|Q9NPP6 | Immunoglobulin heavy chain variant (Fragment) | 1.857 | 1.055 |
| 440 | tr\|Q9UL92\|Q9UL92 | Myosin-reactive immunoglobulin heavy chain variable region (Fragment) | 1.609 | 0.780 |
| 441 | tr\|S6AWE6\|S6AWE6 | IgG L chain | 1.624 | 1.127 |
| 442 | TRA1 | Tumor rejection antigen (Gp96) 1 | 0.421 | 1.011 |
| 443 | TTC38 | Tetratricopeptide repeat protein 38 | 0.103 | 0.771 |
| 444 | TTPA | Alpha-tocopherol transfer protein | 0.130 | — |
| 445 | TUBA4A | Tubulin alpha-4A chain | 0.294 | 0.972 |
| 446 | TUBB | Tubulin beta chain | 0.267 | 1.269 |
| 447 | TUBB1 | Tubulin beta-1 chain | 2.286 | — |
| 448 | TUBB2C | Tubulin, beta 2C | 0.089 | 0.957 |
| 449 | TXN | Isoform 2 of Thioredoxin | 0.138 | 0.916 |
| 450 | UBA1 | Ubiquitin-like modifier-activating enzyme 1 | 0.275 | 0.921 |
| 451 | UBE2N | Ubiquitin-conjugating enzyme E2 N | 0.359 | 0.866 |
| 452 | UGDH | UDP-glucose 6-dehydrogenase | 0.119 | — |
| 453 | UGT2B4 | UDP-glucuronosyltransferase 2B4 | 0.033 | — |
| 454 | UQCRC2 | Cytochrome b-c1 complex subunit 2, mitochondrial | 0.293 | 1.187 |
| 455 | UROC1 | Isoform 2 of Urocanate hydratase | — | 0.597 |
| 456 | V<kappa>1 | Rheumatoid factor C6 light chain (Fragment) | 1.589 | 1.025 |
| 457 | V2-7 | V2-7 protein (Fragment) | 1.751 | 1.109 |
| 458 | VAPB | Vesicle-associated membrane protein-associated protein B/C | 0.330 | 0.929 |
| 459 | VASP | Vasodilator-stimulated phosphoprotein | 2.349 | 1.122 |
| 460 | VAX2 | Ventral anterior homeobox 2 | 3.132 | 1.442 |
| 461 | VCP | Transitional endoplasmic reticulum ATPase | 0.242 | 0.856 |
| 462 | VDAC3 | Voltage-dependent anion-selective channel protein 3 | 0.161 | 1.019 |
| 463 | VIM | Vimentin | 0.131 | 0.720 |
| 464 | YWHAZ | 14-3-3 protein zeta/delta | 0.346 | 1.268 |
