# Supplementary material for: Plasma proteomic study of acute mountain sickness susceptible and resistant individuals
Source: Sci Rep. 2018 Jan 19;8:1265. doi: 10.1038/s41598-018-19818-9 (PMC5775437; doi:10.1038/s41598-018-19818-9)
Supplement: Supplementary file 2 — Supplementary table 2 [file 41598_2018_19818_MOESM2_ESM.docx]

Supplementary Table 2. Changes in KEGG pathwways after acute exposure to high altitude

| **Pathway ID** | **Pathway** | **AMS-HA/ AMS-BL** | | **AMS+HA/ AMS+BL** | |
| --- | --- | --- | --- | --- | --- |
|  |  | **Pvalue** | **Proteins** | **Pvalue** | **Proteins** |
| ko00010 | Glycolysis / Gluconeogenesis | 0.0915 | ALDH1B, ALDH7A1, AKR1A1, DLD, FBP1, PGAM1, ALDH8A1, ALDH9A1, GALM, ALDOA, PGK1,ENO3, PDHA1 | 0.5814 | FBP1, GALM |
| ko00020 | Citrate cycle (TCA cycle) | 0.0000 | SUCLG2, IDH1, MDH2, DLD,OGDH, ACLY, ACON, SDHB, PDHA1, IDH2, DLST, IDH3A, IDH3B, SUCLA2, SUCLG1, SDHA | 0.7469 | SUCLG2 |
| ko00030 | Pentose phosphate pathway | 0.3042 | ALDOA, HIBADH, FBP1, RGN, TKT, TALDO1 | 0.2583 | FBP1, RGN |
| ko00040 | Pentose and glucuronate interconversions | 0.0535 | ALDH1B, UGP2, UGT2B4, UGDH | **—** | — |
| ko00051 | Fructose and mannose metabolism | 0.1796 | ALDOA, FBP1, AKR1C1, KHK | 0.3973 | FBP1 |
| ko00052 | Galactose metabolism | 0.8406 | UGP2 | — | — |
| ko00053 | Ascorbate and aldarate metabolism | 0.0020 | ALDH1B,ALDH7A1, ALDH8A1, ALDH9A1, UGT2B4, RGN, UGDH | 0.3973 | RGN |
| ko00061 | Fatty acid biosynthesis | 0.5444 | FASN | — | — |
| ko00062 | Fatty acid elongation | 0.2337 | Hadhb,TECR, ECHS1, HADHA | 0.4271 | ACAA2 |
| ko00071 | Fatty acid metabolism | 0.0628 | ALDH1B, ALDH7A1, Hadhb, ACADL, ALDH8A1, ALDH9A1, ACAA1, CYP4A11, ECHS1, ACADM, HADHA, CYP4Z1, ACADVL | 0.1051 | ACAA2, ACADS, ACOX1, CYP4Z1 |
| ko00072 | Synthesis and degradation of ketone bodies | — | — | 0.2233 | HMGCS2 |
| ko00120 | Primary bile acid biosynthesis | 0.0836 | SCP2, AKR1C1,HSD17B4 | — | — |
| ko00130 | Ubiquinone and other terpenoid-quinone biosynthesis | 0.6495 | Hpd | 0.183 | Hpd |
| ko00140 | Steroid hormone biosynthesis | 0.0879 | HSD11B1, UGT2B4,AKR1C1,COMT | 0.3329 | HSD11B1 |
| ko00190 | Oxidative phosphorylation | 0.5960 | NDUFAB1, NDUFV2,SDHB, NDUFA4, ATP5C1, ATP6V1B2, ATP5H, UQCRC2, NDUFS1, UQCRB, SDHA | 0.7064 | ATP5C1, NDUFA4 |
| ko00230 | Purine metabolism | 0.9006 | NME2, AK2, CSDE1, ATIC | — | — |
| ko00240 | Pyrimidine metabolism | 0.7506 | AK3, NME2, DPYD | — | — |
| ko00250 | Alanine, aspartate and glutamate metabolism | 0.1076 | GOT2, GIG18, NIT2, ABAT, ASL, GPT2 | 0.5326 | ABAT |
| ko00260 | Glycine, serine and threonine metabolism | 0.0731 | ALDH7A1, SARDH, DLD, PGAM1, DMGDH, SHMT1, CHDH | 0.0475 | BHMT, CHDH, DMGDH |
| ko00270 | Cysteine and methionine metabolism | 0.7600 | GOT2, GIG18 | 0.0987 | AHCY, BHMT |
| ko00280 | Valine, leucine and isoleucine degradation | 0.0265 | ALDH1B, ALDH7A1, DBT, Hadhb, HIBADH, DLD, ALDH6A1, ALDH8A1, ALDH9A1, ACAA1, ECHS1, ACADM, HADHA, IVD, ABAT | 0.0434 | ACOX1, ACAA2, HMGCS2, ACADS, ABAT |
| ko00290 | Valine, leucine and isoleucine biosynthesis | 0.4078 | PDHA1 | — | — |
| ko00300 | Lysine biosynthesis | 0.2304 | ALDH7A1 | — | — |
| ko00310 | Lysine degradation | 0.0073 | ALDH1B, ALDH7A1, OGDH, ALDH8A1, ALDH9A1, ECHS1, HADHA, BBOX1, DLST, TMPO | — | — |
| ko00330 | Arginine and proline metabolism | 0.0703 | ALDH1B, GOT2, ALDH7A1, OAT, GIG18, ALDH8A1, ALDH9A1, LAP3, AGMAT, ASL | — | — |
| ko00340 | Histidine metabolism | 0.1585 | ALDH1B, ALDH7A1, ALDH8A1, ALDH9A1, HAL | 0.4825 | UROC1 |
| ko00350 | Tyrosine metabolism | 0.5223 | GOT2, GIG18, Hpd, COMT | 0.5558 | Hpd |
| ko00360 | Phenylalanine metabolism | 0.0567 | GOT2, GIG18, Hpd, PRDX6, PAH | 0.0834 | Hpd, PRDX6 |
| ko00380 | Tryptophan metabolism | 0.1244 | ALDH1B,ALDH7A1, OGDH, ALDH8A1, ALDH9A1, ECHS1, HADHA | — | — |
| ko00400 | Phenylalanine, tyrosine and tryptophan biosynthesis | 0.0122 | GOT2, GIG18, PAH | — | — |
| ko00410 | beta-Alanine metabolism | 0.0073 | ALDH1B, ALDH7A1, ALDH6A1, ALDH8A1, ALDH9A1, ECHS1, ACADM, HADHA, ABAT, DPYD | 0.2583 | ACOX1, ABAT |
| ko00460 | Cyanoamino acid metabolism | 0.4078 | SHMT1 | — | — |
| ko00480 | Glutathione metabolism | 0.4537 | IDH1,HIBADH, GCLC, LAP3, IDH2 | 0.6189 | GSTA2 |
| ko00500 | Starch and sucrose metabolism | 0.4666 | UGP2, UGT2B4, UGDH, AMY2B | — | — |
| ko00510 | N-Glycan biosynthesis | 0.2337 | STT3A, RPN1, DDOST, GANAB | — | — |
| ko00514 | Other types of O-glycan biosynthesis | 0.7304 | UGT2B4 | — | — |
| ko00520 | Amino sugar and nucleotide sugar metabolism | 0.7096 | UGP2, UGDH | — | — |
| ko00561 | Glycerolipid metabolism | 0.0015 | ALDH1B, ALDH7A1, LPL, AKR1A1, ALDH8A1, ALDH9A1, GK, DAK | — | — |
| ko00562 | Inositol phosphate metabolism | 0.9059 | ALDH6A1 | — | — |
| ko00563 | Glycosylphosphatidylinositol(GPI)-anchor biosynthesis | 0.7304 | GPLD1 | 0.2233 | GPLD1 |
| ko00564 | Glycerophospholipid metabolism | 0.6507 | GPD1, GPD2 | — | — |
| ko00590 | Arachidonic acid metabolism | 0.3510 | CYP2C9, AKR1C1, CYP4A11, CYP2E1, CYP4Z1 | 0.2026 | CYP2C9, CYP4Z1 |
| ko00591 | Linoleic acid metabolism | 0.2036 | CYP2C9, AKR1C1,CYP2E1 | 0.2982 | CYP2C9 |
| ko00592 | alpha-Linolenic acid metabolism | 0.5826 | ACAA1, ACADM | 0.3329 | ACOX1 |
| ko00620 | Pyruvate metabolism | 0.0879 | ALDH1B, ALDH7A1, MDH2, DLD, ALDH8A1, ALDH9A1, GLO1, HAGH, PDHA1, STARD10 | 0.7596 | STARD10 |
| ko00630 | Glyoxylate and dicarboxylate metabolism | 0.7506 | MDH2, ACON, SHMT1 | — | — |
| ko00640 | Propanoate metabolism | 0.0051 | ALDH1B, ALDH7A1, SUCLG2, ALDH6A1, ALDH8A1, ALDH9A1, ECHS1, ACADM, HADHA, ABAT, SUCLA2, SUCLG1 | 0.1215 | ACOX1, ABAT, SUCLG2 |
| ko00650 | Butanoate metabolism | 0.2998 | AKR1C1, ECHS1, HADHA,PDHA1, ABAT | 0.0405 | ABAT, HMGCS2, ACADS |
| ko00670 | One carbon pool by folate | 0.2036 | MTHFD1, SHMT1, ATIC | — | — |
| ko00740 | Riboflavin metabolism | 0.5444 | ACP5 | — | — |
| ko00770 | Pantothenate and CoA biosynthesis | 0.6495 | DPYD | — | — |
| ko00790 | Folate biosynthesis | 0.5444 | QDPR | — | — |
| ko00830 | Retinol metabolism | 0.1554 | CYP2C9, HSD17B11, UGT2B4, CYP4A11, ALDH1A1, DHRS4L1, CYP4Z1 | 0.2583 | CYP2C9, CYP4Z1 |
| ko00860 | Porphyrin and chlorophyll metabolism | 0.3443 | ALAD, UGT2B4, CPOX | 0.0689 | ALAD, CP |
| ko00900 | Terpenoid backbone biosynthesis | — | — | 0.2233 | HMGCS2 |
| ko00920 | Sulfur metabolism | 0.4078 | SULT1A3 | 0.0961 | SULT1A3 |
| ko00970 | Aminoacyl-tRNA biosynthesis | 0.8738 | TARS, ALB, AFM | 0.6378 | IARS2 |
| ko00980 | Metabolism of xenobiotics by cytochrome P450 | 0.5970 | HSD11B1, CYP2C9, UGT2B4, AKR1C1, CYP2E1 | 0.0903 | GSTA2, HSD11B1, CYP2C9 |
| ko00982 | Drug metabolism - cytochrome P450 | 0.7826 | CYP2C9, UGT2B4, CYP2E1,FMO1 | 0.2956 | GSTA2, CYP2C9 |
| ko00983 | Drug metabolism - other enzymes | 0.7078 | UGT2B4, CES1, DPYD |  |  |
| ko01040 | Biosynthesis of unsaturated fatty acids | 0.2337 | ACAA1, TECR, ACADM, HADHA | 0.4271 | ACOX1 |
| ko01100 | Metabolic pathways | 0.0011 | ALAD, ALDOA, ALDH1B, GOT2, ALDH7A1, OAT, GIG18, SUCLG2, HSD11B1, CYP2C9, NDUFAB1, SARDH, AKR1A1, UGP2, DBT, Hadhb, IDH1, MDH2, NDUFV2, ACADL, HIBADH, DLD, STT3A, OGDH, FBP1, PGAM1, ALDH6A1, ALDH8A1, ACLY, ALDH9A1, UGT2B4, ACAA1, SCP2, ACON, AKR1C1, CPOX, CYP4A11, SDHB, Hpd, NDUFA4,ALDH1A1, DHRS4L1, FASN, ATP5C1, GK, ATP6V1B2, MTHFD1, PGK1, DMGDH, GCLC, ENO3, ECHS1, ACADM, HADHA, RPN1, LAP3, HAL, IVD, PDHA1, ATP5H, UQCRC2, IDH2, NDUFS1, RGN, DLST, COMT, CYP2E1, NME2, PRDX6, ABAT, AGMAT, AK2, CYP4Z1, UQCRB, IDH3A, ASL, HSD17B4, DAK, SHMT1, QDPR, CES1, UGDH, IDH3B, TKT, DDOST, SUCLA2, AMY2B, ACADVL, SUCLG1, GPT2, PAH, KHK, DPYD, SDHA, TALDO1, CSDE1, GANAB, ATIC | 0.116 | ABAT, ALAD, ATP5C1, DMGDH, BHMT, SUCLG2, HSD11B1, CYP2C9, Hpd, PRDX6, NDUFA4, CYP4Z1,FBP1, COX1, UROC1, ACAA2, HMGCS2, AHCY, PON3, RGN, ACADS, |
| ko02010 | ABC transporters | — | — | 0.2617 | ABCD3 |
| ko03008 | Ribosome biogenesis in eukaryotes | 0.9949 | RAN |  |  |
| ko03010 | Ribosome | 0.0001 | RPS27A, RPL18A,RPL11,RPSA, RPL15, RPS27L, RPL4, RPS24, RPS26, RPS10,RPL32, RPS2, RPL24, RPL10A, RPS17L, RPS3A, RPL30, RPL7A, RPL27, RPL5, RPS25, RPS16, RPL3, RPL14, RPS8, RPS23, RPL9, RPS14 | 0.4222 | RPS2, RPS9, RPS24, RPL15 |
| ko03013 | RNA transport | 0.7723 | SEC13, ALYREF, KPNB1, PABPC1, Eif4a1, EIF3B, FXR1, EIF3A, RAN, SAP18 | — | — |
| ko03015 | mRNA surveillance pathway | 0.5162 | NUDT21, ALYREF, PPP2CA, PPP2R1A, PABPC1, TARDBP, SAP18 | — | — |
| ko03018 | RNA degradation | 0.9709 | ENO3, PABPC1 | — | — |
| ko03030 | DNA replication | 0.9059 | PCNA | — | — |
| ko03040 | Spliceosome | 0.7468 | SNRPG, SF3B3, ALYREF, DHX15, DDX5, PCBP1, RBMX, Hnrnpk, G3BP, SNRNP200, SNRPE, SNRPD3, SRSF1 | — | — |
| ko03050 | Proteasome | 0.0071 | PSMC3, PSMA3, PSMD12, PSMD1, PSMD2, PSMD11, PSMA7, PSMC4, PSMB1, PSMA2, PSMA1, PSMC5, PSMA6, PSMD7 | 0.8043 | PSMD12 |
| ko03060 | Protein export | 0.7096 | SRP68, HSPA5 | — | — |
| ko03320 | PPAR signaling pathway | 0.2728 | SLC27A2, LPL, BRL, ACADL, APOA5, ACAA1, SCP2, CYP4A11, GK, ACADM, CYP4Z1 | 0.1217 | ACOX1, HMGCS2, BRL, CYP4Z1 |
| ko03410 | Base excision repair | 0.1346 | HMGB1, PCNA | — | — |
| ko03420 | Nucleotide excision repair | 0.2730 | DDB1, RAD23B, PCNA | — | — |
| ko03430 | Mismatch repair | 0.7304 | PCNA | — | — |
| ko04010 | MAPK signaling pathway | 0.8558 | Rap1a, CPNE3, FLNB, ITIH3, EGFR | 0.7831 | ITIH1 |
| ko04012 | ErbB signaling pathway | 0.2730 | PA2G4, CPNE3, EGFR | — | — |
| ko04013 | MAPK signaling pathway - fly | 0.2289 | EGFR, TPM3 | — | — |
| ko04020 | Calcium signaling pathway | 0.8524 | VDAC3, PA2G4, IGHA2, IGHA1, SLC25A6, CPNE3, EGFR, IGHG1, IGHM, CALM2, tr\|A2N011\|A2N011_HUMAN | — | — |
| ko04060 | Cytokine-cytokine receptor interaction | 0.0879 | INHBC, PF4, EGFR, PPBP | 0.0553 | PF4, PPBP |
| ko04062 | Chemokine signaling pathway | 0.4026 | Rap1a, PF4, CPNE3, GNB2, PPBP | 0.221 | PF4, PPBP |
| ko04064 | NF-kappa B signaling pathway | 0.9831 | IGHA2, IGHA1, CPNE3, IGHG1, IGHM, tr\|A2N011\|A2N011_HUMAN | — | — |
| ko04070 | Phosphatidylinositol signaling system | 0.6507 | CPNE3, CALM2 | — | — |
| ko04110 | Cell cycle | 0.8946 | YWHAG, YWHAZ, PCNA | — | — |
| ko04114 | Oocyte meiosis | 0.2025 | YWHAG, PPP2CA, PPP2R1A, YWHAZ, CALM2 | — | — |
| ko04115 | p53 signaling pathway | 0.9852 | THBS1 | 0.0405 | THBS1, CYCS, COMP |
| ko04120 | Ubiquitin mediated proteolysis | 0.5035 | TCEB1, DDB1, UBA1, UBE2N, TMPO | — | — |
| ko04141 | Protein processing in endoplasmic reticulum | 0.1593 | SEC13, DNAJC3, VCP, STT3A, SSR1, SSR3, RPN1, HSP90AA1, RAD23B, DDOST, TRA1, HSPA5, PDIA6, TMPO, PDIA4, GANAB, LMAN1 | — | — |
| ko04142 | Lysosome | 0.6399 | AP1B1, COPB1, CLTA, ACP5, NUCB1 | 0.6893 | CTSB |
| ko04144 | Endocytosis | 0.3055 | PA2G4, OPA1, ARF6, Ap2m1, EGFR, AP2A2, EPN1, CLTA, RAB11A, TFRC | 0.8407 | ARF6 |
| ko04145 | Phagosome | 0.9435 | HPR, TUBB2C, IGHA2, IGHA1, C1RL, ATP6V1B2, TUBB, ACTR3, IGHG1, ITGB1, IGHM,TUBA4A, THBS1, TFRC, tr\|A2N011\|A2N011_HUMAN | 0.6929 | THBS1, COMP, C1R, C1RL |
| ko04146 | Peroxisome | 0.1814 | SLC27A2, IDH1, ACAA1, SCP2, DHRS4L1, PRDX1, ACADM, CROT, IDH2, HSD17B4 | 0.2063 | ACOX1, ABCD3, HACL1 |
| ko04210 | Apoptosis | 0.8027 | AIFM1, PRKAR2B | 0.4555 | CYCS |
| ko04260 | Cardiac muscle contraction | 0.9963 | UQCRC2, UQCRB | 0.7831 | ITIH1 |
| ko04270 | Vascular smooth muscle contraction | 0.6697 | CYP4A11, CPNE3,CYP4Z1, CALM2 | 0.6189 | CYP4Z1 |
| ko04310 | Wnt signaling pathway | 0.6058 | PPP2CA, PPP2R1A, CPNE3 | — | — |
| ko04320 | Dorso-ventral axis formation | 0.6495 | EGFR | — | — |
| ko04350 | TGF-beta signaling pathway | 0.0532 | INHBC, FBLN1, PPP2CA,PPP2R1A, THBS1 | 0.1843 | THBS1, COMP |
| ko04360 | Axon guidance | 0.6596 | LASP1, FES, ITGB1 | — | — |
| ko04370 | VEGF signaling pathway | 0.8775 | CPNE3 | — | — |
| ko04380 | Osteoclast differentiation | 0.7927 | ACP5 | — | — |
| ko04510 | Focal adhesion | 0.7397 | ACTN1, ACTN4, Rap1a, COL6A2, CPNE3, FLNB, EGFR, ACTR3, VASP, COL4A2, ITGB1, THBS1 | 0.1611 | ACTN1, TLN1, THBS1, RELN, COMP |
| ko04512 | ECM-receptor interaction | 0.9160 | COL6A2, COL4A2, ITGB1, THBS1 | 0.1562 | THBS1, RELN, COMP |
| ko04514 | Cell adhesion molecules (CAMs) | 0.5750 | CPNE3, CDH1, ITGB1, CDH5 | 0.5779 | CDH1 |
| ko04520 | Adherens junction | 0.2264 | ACTN1, ACTN4, EGFR, IQGAP2, ACTR3, CDH1, CTNNA1 | 0.2956 | ACTN1, CDH1 |
| ko04530 | Tight junction | 0.4899 | ACTN1, ACTN4, SPTAN1, PPP2CA, PPP2R1A, PLS3, CPNE3, ACTR3, CTNNA1 | 0.8487 | ACTN1 |
| ko04540 | Gap junction | 0.2998 | TUBB2C, CPNE3, TUBB, EGFR, TUBA4A | — | — |
| ko04610 | Complement and coagulation cascades | 0.4999 | SERPINA10, HPR, C7,SERPINA6, C8A, C5, C1RL, C4B, C8G, CFHR2, C6, F10, CFHR1, C4A, CFH, F9, C8B, KLKB1, MASP1 | 3E-07 | AMBP, C1QB, MASP1, C1R, C4B, C7, CPB2, KNG1, C1QC, C8G, CFHR2, C6, C1S, CFHR1, C1RL, MASP1, C4A |
| ko04612 | Antigen processing and presentation | 0.8494 | HSP90AA1,HSPA4, HSPA5 | 0.6189 | CTSB |
| ko04621 | NOD-like receptor signaling pathway | 0.2730 | PACSIN3, HSP90AA1, TRA1 | 0.3329 | GVINP1 |
| ko04622 | RIG-I-like receptor signaling pathway | 0.5444 | DAK | — | — |
| ko04640 | Hematopoietic cell lineage | 0.9767 | IGHA2, IGHA1, IGHG1, IGHM, TFRC, tr\|A2N011\|A2N011_HUMAN | — | — |
| ko04650 | Natural killer cell mediated cytotoxicity | 0.9767 | IGHA2, IGHA1, CPNE3, IGHG1, IGHM, tr\|A2N011\|A2N011_HUMAN | — | — |
| ko04662 | B cell receptor signaling pathway | 0.9629 | IGHA2, IGHA1, CPNE3, IGHG1, IGHM, tr\|A2N011\|A2N011_HUMAN | — | — |
| ko04664 | Fc epsilon RI signaling pathway | 0.9682 | IGHA2, IGHA1, CPNE3, IGHG1, IGHM, tr\|A2N011\|A2N011_HUMAN | — | — |
| ko04666 | Fc gamma R-mediated phagocytosis | 0.9240 | OPA1, ARF6, IGHA2, IGHA1, CPNE3, IGHG1, IGHM, ARPC2, VASP, tr\|A2N011\|A2N011_HUMAN | 0.9666 | ARF6 |
| ko04670 | Leukocyte transendothelial migration | 0.0632 | ACTN1, ACTN4, MMP2, Rap1a, MSN, CPNE3, ACTR3, CTNNA1, VASP, ITGB1, CDH5 | 0.7831 | ACTN1 |
| ko04672 | Intestinal immune network for IgA production | 0.9759 | IGHA2, IGHA1, IGHG1, IGHM, tr\|A2N011\|A2N011_HUMAN | — | — |
| ko04720 | Long-term potentiation | 0.4149 | Rap1a, CPNE3, CALM2 | — | — |
| ko04721 | Synaptic vesicle cycle | 0.2500 | OPA1, ATP6V1B2, Ap2m1, AP2A2, CLTA | — | — |
| ko04722 | Neurotrophin signaling pathway | 0.2910 | Rap1a, YWHAG, YWHAZ, CALM2 | — | — |
| ko04723 | Retrograde endocannabinoid signaling | 0.6507 | CPNE3, GNB2 | — | — |
| ko04724 | Glutamatergic synapse | 0.8683 | CPNE3, GNB2 | — | — |
| ko04725 | Cholinergic synapse | 0.6507 | CPNE3, GNB2 | — | — |
| ko04726 | Serotonergic synapse | 0.4666 | CYP2C9, CPNE3, GNB2, CYP4Z1 | 0.1664 | CYP2C9, CYP4Z1 |
| ko04727 | GABAergic synapse | 0.2730 | CPNE3, GNB2, ABAT | 0.3329 | ABAT |
| ko04728 | Dopaminergic synapse | 0.2596 | PPP2CA, PPP2R1A, CPNE3, GNB2, COMT, CALM2 | — | — |
| ko04730 | Long-term depression | 0.4828 | PPP2CA, PPP2R1A, CPNE3 | — | — |
| ko04740 | Olfactory transduction | 0.5444 | CALM2 | — | — |
| ko04744 | Phototransduction | 0.4078 | CALM2 | — | — |
| ko04745 | Phototransduction - fly | 0.2730 | CPNE3, ACTR3, CALM2 | — | — |
| ko04810 | Regulation of actin cytoskeleton | 0.8654 | ACTN1, ACTN4, MSN, RDX, EGFR, IQGAP2, ACTR3, ARPC2, ITGB1 | 0.9268 | ACTN1 |
| ko04910 | Insulin signaling pathway | 0.8126 | FBP1, FASN,PRKAR2B, CALM2 | 0.6893 | FBP1 |
| ko04912 | GnRH signaling pathway | 0.4666 | MMP2, CPNE3, EGFR, CALM2 | — | — |
| ko04914 | Progesterone-mediated oocyte maturation | 0.9059 | HSP90AA1 | — | — |
| ko04916 | Melanogenesis | 0.7096 | CPNE3, CALM2 | — | — |
| ko04960 | Aldosterone-regulated sodium reabsorption | 0.8775 | CPNE3 | — | — |
| ko04961 | Endocrine and other factor-regulated calcium reabsorption | 0.2169 | OPA1, Ap2m1, CPNE3, AP2A2, CLTA, RAB11A | — | — |
| ko04962 | Vasopressin-regulated water reabsorption | 0.6507 | DCTN2, RAB11A | — | — |
| ko04964 | Proximal tubule bicarbonate reclamation | 0.8027 | CRP, APCS | 0.1148 | CRP, APCS |
| ko04966 | Collecting duct acid secretion | 0.4149 | CRP, ATP6V1B2, APCS | 0.0834 | CRP, APCS |
| ko04970 | Salivary secretion | 0.2998 | CPNE3, CST3, AMY2B, CD5L, CALM2 | — | — |
| ko04971 | Gastric acid secretion | 0.5035 | CRP, CPNE3, APCS, ACTR3, CALM2 | 0.2583 | CRP, APCS |
| ko04972 | Pancreatic secretion | 0.5323 | Rap1a, CRP, CPNE3, APCS, AMY2B, RAB11A | 0.1215 | CPB2, CRP, APCS |
| ko04973 | Carbohydrate digestion and absorption | 0.7096 | CPNE3, AMY2B | — | — |
| ko04974 | Protein digestion and absorption | 0.7882 | COL6A2, COL4A2, DPP4 | 0.5779 | CPB2 |
| ko04975 | Fat digestion and absorption | 0.9444 | GOT2 | — | — |
| ko04976 | Bile secretion | 0.8210 | CRP, UGT2B4, APCS | 0.221 | CRP, APCS |
| ko05010 | Alzheimer's disease | 0.5853 | NDUFAB1, LPL,NDUFV2, SDHB, APOE, NDUFA4, ATP5C1, ATP5H, UQCRC2, NDUFS1, UQCRB, TMPO, SDHA, CALM2 | 0.5986 | ATP5C1, CYCS, NDUFA4 |
| ko05012 | Parkinson's disease | 0.1752 | VDAC3, NDUFAB1, NDUFV2, SDHB, SEPT2, NDUFA4, SLC25A6, ATP5C1, UBA1, ATP5H, UQCRC2, NDUFS1, UQCRB, SEPT7, SDHA | 0.4642 | ATP5C1, CYCS, NDUFA4 |
| ko05016 | Huntington's disease | 0.3164 | VDAC3, NDUFAB1, NDUFV2, SDHB, NDUFA4, DCTN2, SLC25A6, ATP5C1, Ap2m1,PACSIN3, ATP5H, UQCRC2, NDUFS1, AP2A2, UQCRB, CLTA, SDHA | 0.6313 | ATP5C1, CYCS, NDUFA4 |
| ko05020 | Prion diseases | 0.0358 | C7, C8A, C5, CPNE3, C8G, C6, C8B, HSPA5 | 0.0013 | C7, C1QB, C1QC, C8G, C6 |
| ko05031 | Amphetamine addiction | 0.7600 | CPNE3, CALM2 | — | — |
| ko05032 | Morphine addiction | 0.4190 | CPNE3, GNB2 | — | — |
| ko05034 | Alcoholism | 0.9815 | GNB2, CALM2 | — | — |
| ko05100 | Bacterial invasion of epithelial cells | 0.4330 | OPA1, ACTR3, CDH1, ARPC2, CLTA, ITGB1 | 0.7469 | CDH1 |
| ko05110 | Vibrio cholerae infection | 0.5223 | ATP6V1B2, CPNE3, ACTR3, PDIA4 | — | — |
| ko05120 | Epithelial cell signaling in Helicobacter pylori infection | 0.7096 | ATP6V1B2, EGFR | — | — |
| ko05130 | Pathogenic Escherichia coli infection | 0.2724 | YWHAG, TUBB2C, YWHAZ, CPNE3, TUBB, ACTR3, CDH1, TUBA4A, ARPC2, ITGB1 | 0.5218 | KRT10, CDH1 |
| ko05131 | Shigellosis | 0.8494 | ACTR3, ARPC2, ITGB1 | — | — |
| ko05132 | Salmonella infection | 0.9504 | FLNB, ACTR3, ARPC2 | — | — |
| ko05133 | Pertussis | 0.5562 | HPR, C5, C1RL, C4B, C4A, CALM2, ITGB1 | 0.0004 | C1QB, C1R, C4B, C1QC, C1S, C1RL, C4A |
| ko05134 | Legionellosis | 0.9300 | VCP, RAB1A | 0.5779 | CYCS |
| ko05140 | Leishmaniasis | 0.9387 | IGHA2, IGHA1, CPNE3, IGHG1, IGHM, ITGB1, tr\|A2N011\|A2N011_HUMAN | — | — |
| ko05142 | Chagas disease (American trypanosomiasis) | 0.9300 | PPP2CA, PPP2R1A | 0.2026 | C1QC, C1QB |
| ko05143 | African trypanosomiasis | 0.9653 | HPR, IGHA2, IGHA1, CPNE3, IGHG1, IGHM, tr\|A2N011\|A2N011_HUMAN | 0.5279 | HBA1, HBB |
| ko05144 | Malaria | 0.9672 | THBS1 | 0.0002 | THBS1,HBA1, COMP, HBB |
| ko05145 | Toxoplasmosis | 0.9852 | ITGB1 | 0.5558 | CYCS |
| ko05146 | Amoebiasis | 0.8418 | ACTN1, ACTN4, IGHA2, C8A, IGHA1, CPNE3, SERPINB8, C8G, IGHG1, IGHM, C8B, COL4A2, tr\|A2N011\|A2N011_HUMAN | 0.8902 | ACTN1, C8G |
| ko05150 | Staphylococcus aureus infection | 0.9371 | HPR, IGHA2, IGHA1, C5, C1RL, C4B, CFHR2, IGHG1, CFHR1, C4A, IGHM, CFH, MASP1, tr\|A2N011\|A2N011_HUMAN | 0.0015 | C1R, C4B, C1QC, CFHR2, C1S, CFHR1, C1RL, , C4A, C1QB, KRT10, MASP1 |
| ko05152 | Tuberculosis | 0.9979 | IGHA2, IGHA1, IGHG1, IGHM, CALM2, tr\|A2N011\|A2N011_HUMAN | 0.9683 | CYCS |
| ko05160 | Hepatitis C | 0.2910 | PPP2CA, PPP2R1A, EGFR, TMPO | — | — |
| ko05162 | Measles | 0.9778 | MSN, IGHA2, IGHA1, IGHG1, IGHM , TMPO, tr\|A2N011\|A2N011_HUMAN | — | — |
| ko05164 | Influenza A | 0.8076 | DNAJC3, KPNA3, SLC25A6, CPNE3, ACTR3, TMPO | 0.8141 | CYCS |
| ko05166 | HTLV-I infection | 0.9651 | VDAC3, SLC25A6 , RAN, PCNA | 0.8141 | TLN1 |
| ko05168 | Herpes simplex infection | 0.6537 | ALYREF, C5, Hnrnpk, TMPO, CFP, SRSF1 | 0.4051 | C1QBP, CYCS |
| ko05169 | Epstein-Barr virus infection | 0.5241 | PSMC3, PSMD12, PSMD1, YWHAG, PSMD2, PSMD11, IGHA2, IGHA1, YWHAZ, PSMC4, SND1, VIM, IGHG1, PSMC5, IGHM, PSMD7, TMPO, RAN, tr\|A2N011\|A2N011_HUMAN | 0.9884 | PSMD12 |
| ko05200 | Pathways in cancer | 0.5015 | MMP2, TCEB1, CPNE3, EGFR, ARPC2, ITGB1, HSP90AA1, CDH1, TRA1, COL4A2 | 0.3972 | GSTA2 , CYCS, CDH1 |
| ko05202 | Transcriptional misregulation in cancer | 0.9837 | IGHA2, IGHA1, DDX5, ARPC2, IGHG1, IGHM, tr\|A2N011\|A2N011_HUMAN | — | — |
| ko05210 | Colorectal cancer | — | — | 0.2617 | CYCS |
| ko05211 | Renal cell carcinoma | 0.7096 | Rap1a, TCEB1 | — | — |
| ko05212 | Pancreatic cancer | 0.9277 | EGFR | — | — |
| ko05213 | Endometrial cancer | 0.2730 | EGFR, CDH1, CTNNA1 | 0.3329 | CDH1 |
| ko05214 | Glioma | 0.3443 | CPNE3, EGFR, CALM2 | — | — |
| ko05215 | Prostate cancer | 0.6596 | EGFR, HSP90AA1, TRA1 | 0.5082 | GSTA2 |
| ko05216 | Thyroid cancer | 0.7927 | CDH1 | 0.2617 | CDH1 |
| ko05218 | Melanoma | 0.5826 | EGFR, CDH1 | 0.3329 | CDH1 |
| ko05219 | Bladder cancer | 0.179595 | MMP2, EGFR, CDH1, THBS1 | 0.0108 | CDH1 |
| ko05221 | Acute myeloid leukemia | 0.544353 | ARPC2 | — | — |
| ko05222 | Small cell lung cancer | 0.838446 | COL4A2, ITGB1 | 0.4825 | CYCS |
| ko05223 | Non-small cell lung cancer | 0.582591 | CPNE3, EGFR | — | — |
| ko05310 | Asthma | 0.97154 | IGHA2, IGHA1, IGHG1, IGHM, tr\|A2N011\|A2N011_HUMAN | — | — |
| ko05320 | Autoimmune thyroid disease | 0.982756 | IGHA2, IGHA1, IGHG1, IGHM, tr\|A2N011\|A2N011_HUMAN | — | — |
| ko05322 | Systemic lupus erythematosus | 0.557346 | ACTN1, ACTN4, HPR, C7, IGHA2, C8A, IGHA1, C5, C1RL, C4B, C8G, C6, IGHG1, C4A, IGHM, C8B, SNRPD3, tr\|A2N011\|A2N011_HUMAN | 0.0019 | C1QB, C1R, C4B, C7, ACTN1, C1QC, C8G, C6, C1S, C1RL, C4A |
| ko05323 | Rheumatoid arthritis | 0.959834 | IGHA2, IGHA1, ATP6V1B2, IGHG1, IGHM, ACP5, tr\|A2N011\|A2N011_HUMAN | — | — |
| ko05330 | Allograft rejection | 0.982756 | IGHA2, IGHA1, IGHG1, IGHM, tr\|A2N011\|A2N011_HUMAN | — | — |
| ko05340 | Primary immunodeficiency | 0.954693 | IGHA2, IGHA1, IGHM, IGLC2, IGHG1,IGKV1-5, sp\|P01714\|LV301_HUMAN, sp\|P01625\|KV402_HUMAN, sp\|P01613\|KV121_HUMAN, sp\|P04430\|KV122_HUMAN, sp\|P06311\|KV311_HUMAN, tr\|A2MYD4\|A2MYD4_HUMAN, sp\|P01611\|KV119_HUMAN, tr\|S6AWE6\|S6AWE6_HUMAN, tr\|A2N011\|A2N011_HUMAN, | — | — |
| ko05410 | Hypertrophic cardiomyopathy (HCM) | 0.988261 | ACTR3, ITGB1 | 0.7196 | ITIH1 |
| ko05412 | Arrhythmogenic right ventricular cardiomyopathy (ARVC) | 0.475027 | ACTN1, ACTN4, ARPC2, ACTR3, CTNNA1, DSG2, ITGB1 | 0.1562 | ACTN1, ITIH1, DSG2 |
| ko05414 | Dilated cardiomyopathy | 0.996301 | IGHA2, IGHA1, ACTR3, IGHG1, IGHM, ITGB1, tr\|A2N011\|A2N011_HUMAN | 0.9729 | ITIH1 |
| ko05416 | Viral myocarditis | 0.993846 | IGHA2, IGHA1, ACTR3, IGHG1, IGHM, tr\|A2N011\|A2N011_HUMAN | 0.9566 | CYCS |
